# Integrating a Transdiagnostic Clinical Approach with Whole-Exome Sequencing in Neurodevelopmental Disorders: A Single-Center Exploratory Study

**DOI:** 10.3390/diagnostics16213674

**Published:** 2026-11-06

**Authors:** Emanuele Occhipinti, Ilaria Pedi, Stefano Pagano, Maria Laura Manca, Rosanna Trovato, Roberta Battini, Filippo M. Santorelli

**Affiliations:** 1 Department of Developmental Neuroscience, IRCCS Fondazione Stella Maris, 56128 Pisa, Italy; emanuele.occhipinti@fsm.unipi.it (E.O.); stefanop@hotmail.it (S.P.);; 2 Department of Clinical and Experimental Medicine, University of Pisa, 56126 Pisa, Italy; 3 Department of Health Sciences, University of Milan, 20142 Milan, Italy; 4 Department of Mathematics, University of Pisa, 56127 Pisa, Italy

**Keywords:** neurodevelopmental disorders, transdiagnostic phenotyping, latent class analysis, whole-exome sequencing, variants of uncertain significance, Reactome, pathway burden

## Abstract

**Background/Objectives:** Neurodevelopmental disorders overlap in cognitive, language, motor, behavioral and social-communication features and vary widely in their genetic causes, which limits etiological research and patient stratification. We investigated whether grouping patients by data-driven clinical profiles rather than by diagnosis points to different biological pathways. **Methods:** We retrospectively evaluated a single-center cohort of 128 patients, grouping them into diagnosis and genetics-independent clinical profiles and into six overlapping DSM-5 diagnostic groups. In each group, the genes carrying a causative variant found by whole-exome sequencing were linked to the biological pathways they belong to. Data on variants of uncertain significance (VUS) were available for 113 of the 128 patients, in whom we counted VUS in these pathways. Each group was compared with the other patients by Poisson regression accounting for each patient’s total VUS count. Analyses were repeated in patients with high-confidence profile assignments. **Results:** Four clinical profiles were identified (labeled C1–C4), and 89.1% of patients were assigned to one with high confidence. A pathway was considered enriched in a group when the group’s patients carried more VUS in it than other patients (*p* < 0.05, uncorrected). The profiles yielded one enriched pathway, Transcriptional Regulation by *MECP2* in profile C3 Social–Sensory/Repetitive (IRR 1.46, 95% CI 1.06–2.03; *p* = 0.0223); the diagnoses yielded 21, including the same pathway, which emerged in ADHD rather than in ASD, the diagnosis closest to C3. In the high-confidence subset, Myogenesis appeared in C4 Motor–Neurological, with no diagnostic counterpart. No association survived multiple testing. **Conclusions:** The two groupings proved complementary rather than alternative: both pointed to Transcriptional Regulation by *MECP2*, and each raised hypotheses the other missed. All these hypotheses require independent replication and functional or epigenomic validation.

## 1. Introduction

Neurodevelopmental disorders (NDDs) involve difficulties in cognition, language, motor development, attention, social communication, adaptive functioning, or behavior. Diagnostic categories of the Diagnostic and Statistical Manual of Mental Disorders, Fifth Edition (DSM-5) include autism spectrum disorder (ASD), attention-deficit/hyperactivity disorder (ADHD), communication disorders, intellectual disability (ID), developmental coordination disorder (DCD), and specific learning disorder (SLD) [1,2]. These conditions frequently co-occur and share clinical manifestations. Consequently, the same diagnosis may encompass patients with different symptom profiles, while patients with different diagnoses may share similar developmental difficulties [3,4,5,6,7,8,9,10].

If patients are grouped only by diagnosis, biologically relevant similarities may be diluted by within-category heterogeneity. A transdiagnostic approach can complement categorical diagnoses by identifying latent phenotypic profiles based on shared patterns of developmental, psychiatric, neurological, and behavioral features [5,6,7,8,9]. Such profiles offer a person-centered representation of phenotypic variation.

Latent class analysis (LCA) is a statistical method that assumes a sample is made up of a small number of hidden subgroups and identifies them from shared patterns of features [11,12,13,14,15]. It can reveal recurring combinations of features without assuming that diagnostic labels define the underlying groups. We selected LCA as the exploratory clustering approach for this study because our phenotypic indicators were binary and because the method provides a model-based framework for estimating clinically interpretable subgroups and individual class-membership probabilities, rather than imposing continuous latent dimensions or relying only on pairwise phenotypic similarity [14,15]. Although the resulting profiles may vary across samples, they provide a clinically meaningful framework for generating biological hypotheses that can be tested and validated in independent cohorts [11,14,15].

Genetic heterogeneity creates a parallel problem. Many genes can cause overlapping neurodevelopmental phenotypes, while individual genes may be associated with broad phenotypic spectra. Large sequencing studies nevertheless indicate convergence on biological processes, including transcriptional regulation, chromatin remodeling, neuronal development, and synaptic function [16,17]. Exome or genome sequencing is recommended for many children with developmental delay, ID, or congenital anomalies [18,19], but testing that does not yield a diagnosis often returns variants of uncertain significance (VUS) [20,21,22]. Under American College of Medical Genetics and Genomics/Association for Molecular Pathology (ACMG/AMP) guidance, a VUS does not establish pathogenicity and should not be used for clinical decision-making, and a VUS may be reclassified as further evidence accumulates [23,24,25]. Any VUS-based pathway association must therefore remain hypothesis-generating.

Clinical and genetic heterogeneity are often addressed separately, and studies that link latent phenotypic profiles to genomic data rarely extend the comparison to conventional diagnostic groups and to pathway-level analysis of residual VUS [26,27,28,29]. Phenotypic clustering studies may stop after describing clinical profiles, whereas genetic studies commonly compare diagnosis-defined or gene-defined cohorts. Standardized Human Phenotype Ontology (HPO) encoding [30,31] and curated pathway resources such as Reactome [32] permit an integrated test of whether clinical profiles and conventional diagnoses nominate overlapping or distinct molecular hypotheses.

### Objectives

This study investigates whether the way patients are grouped clinically changes which biological hypotheses emerge from their genetic data. In a cohort of patients with neurodevelopmental disorders, we group them by data-driven clinical profiles, defined independently of diagnostic labels and genetic findings, and look for pathways in which variants of uncertain significance recur. The same analysis is then carried out using DSM-5 diagnoses, so that the two groupings can be compared: if a transdiagnostic view captures something the diagnostic categories do not, it should point to different, or additional, biological hypotheses. Finally, we examine whether a more sharply defined clinical grouping yields more specific pathway signals by repeating the analysis in the patients whose profile assignment is least ambiguous. The aim is not to establish causality, but to define a reproducible way of prioritizing variants for functional or clinical reassessment.

## 2. Materials and Methods

Patients were phenotyped with a standardized checklist and grouped into clinical profiles by latent class analysis; the genes carrying clinically informative exome findings then defined a set of anchor pathways for each profile, in which VUS burden was tested. The same procedure was applied to the DSM-5 diagnostic groups and to the high-confidence profile subset, so that the groupings could be compared in the same patients.

### 2.1. Study Design and Analytical Cohort

The study followed the Declaration of Helsinki, including its 2024 revision. Parents or legal guardians provided written informed consent for genetic testing and the use of clinical and genetic data. The study was approved by the Regione Toscana CEP Ethics Committee (approval code 154/2020; 3 July 2020).

This retrospective, single-center exploration screened 1104 patients referred to our genetic counseling service between January 2024 and February 2026. The study was conducted at IRCCS Fondazione Stella Maris, a pediatric neuropsychiatric research and clinical center providing multidisciplinary assessments for neuropsychiatric disorders, neuroimaging and neurophysiology investigations, and genetic counseling. Of the 1104 screened patients, 521 had undergone both chromosomal microarray analysis (CMA) and whole-exome sequencing (WES) using routine diagnostic methodologies, and 343 of these had completed a clinical genetic evaluation at our center within the selected age range of 6–16 years. Eligibility also required at least one multidisciplinary clinical evaluation at our center between 6 and 16 years of age; additional evaluations outside this age range were permitted. The 6–16-year range was chosen to improve phenotypic comparability, because preschool children may not yet have manifested the full spectrum of developmental, psychiatric, or neurological features considered in the analysis. Manual review by two investigators (E.O. and I.P.) excluded 215 patients for the following mutually exclusive reasons: diagnoses outside the study eligibility criteria, including perinatal brain injury, severe structural brain malformations, or isolated intellectual disability without additional features suggesting a neurodevelopmental disorder (*n* = 60); failure to meet the age criterion (*n* = 80); unavailable WES data (*n* = 17); unavailable clinical data (*n* = 24); WES reports not yet received at data lock (*n* = 31); and a phenotype explained by a pathogenic or likely pathogenic CMA finding (*n* = 3), yielding a final analytical cohort of 128 patients with neurodevelopmental disorders (NDDs).

**Figure 1:**
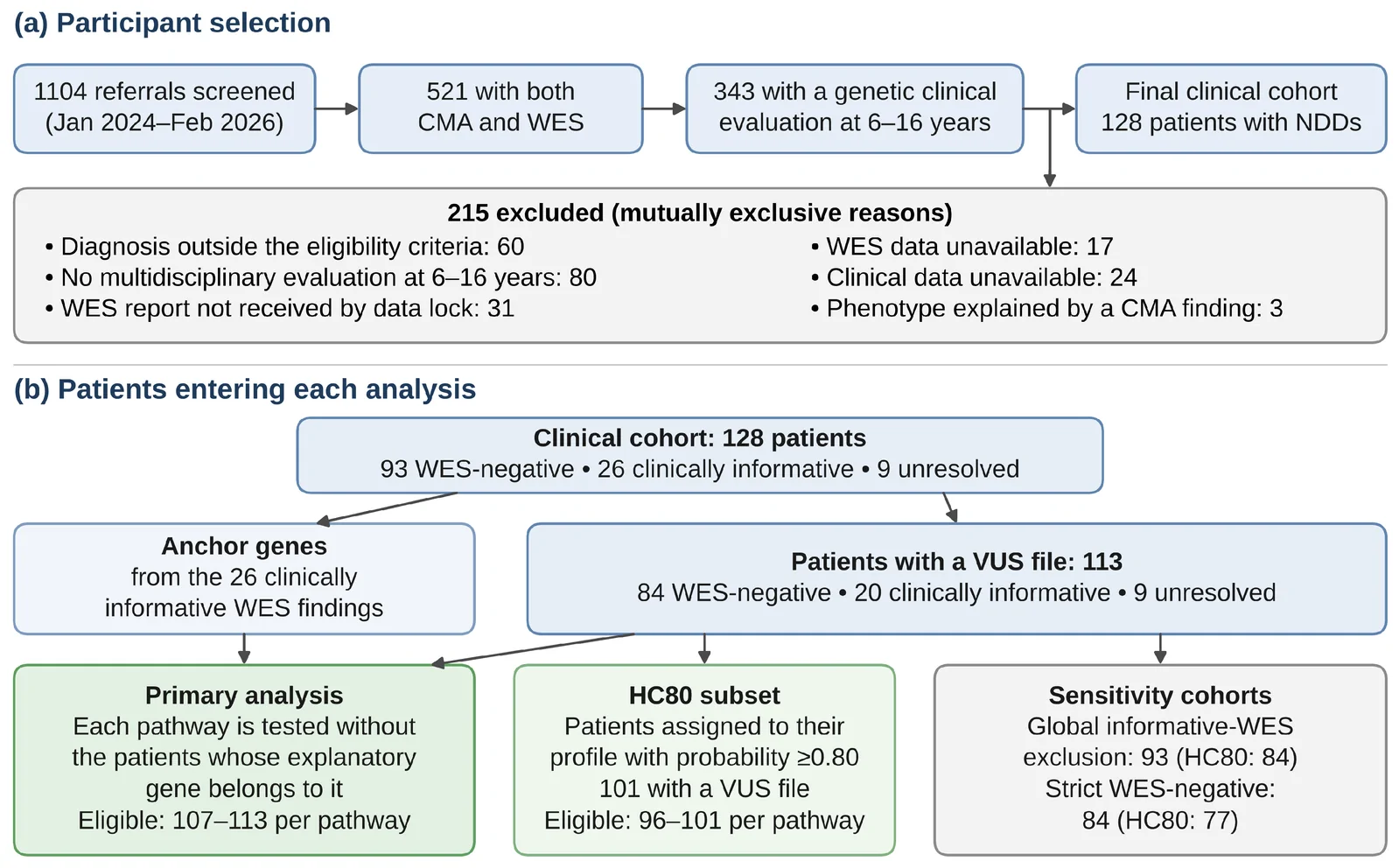
Participant selection and analytical flow. (**a**) Selection of the 128 patients with neurodevelopmental disorders (NDDs) from 1104 referrals; each excluded patient is counted under one reason only. (**b**) Patients entering each analysis. Anchor genes came from the 26 clinically informative whole-exome sequencing (WES) findings of the full cohort. Variant of uncertain significance (VUS) files were available for 113 patients. In the primary analysis, a patient was excluded only from the pathways containing their own explanatory gene, so the number of eligible patients varied between pathways. HC80 is the subset of patients assigned to their profile with a probability of at least 0.80. The global informative-WES exclusion cohort and the strict WES-negative cohort were used for sensitivity analyses.

The study comprised two parallel analytical tracks using the same clinical cohort (Figure 1): diagnosis- and genetics-independent profiles derived through latent class analysis (LCA), and diagnostic groups based on the DSM-5. The cohort included 82 males and 46 females, with age at the last clinical evaluation ranging from 6 to 17 years. WES findings were negative in 93 patients and clinically informative in 26; in the remaining 9, results were available but were still to be evaluated as causative of the clinical phenotype. The genes carrying these 26 clinically informative findings were used as anchor genes: in each group, the pathways containing the group’s anchor genes were the ones tested for VUS. The 9 unresolved cases were retained in the clinical cohort, but their findings were not used as anchor genes. VUS files were available for 113/128 patients: 84 WES-negative, 20 with clinically informative WES findings, and all 9 unresolved cases; 15 patients had no VUS file available. Family structures comprised 124 trios, two father–proband duos, one mother–proband duo, and one singleton. CMA status was recorded as negative for all included patients. Because latent class assignment is probabilistic, every analysis was also repeated in a high-confidence subset (HC80), comprising the 114/128 patients (89.1%) whose maximum posterior class-assignment probability was at least 0.80, that is, those with the least ambiguous clinical profile. The same restriction was applied to the diagnosis-based analyses so that the two frameworks remained comparable.

### 2.2. HPO-Based Deep Phenotyping and Chart Abstraction

Clinical information was retrospectively abstracted from longitudinal medical records and encoded using Human Phenotype Ontology (HPO) terms. For each feature, we selected the lowest, most specific HPO level that still described the symptom clearly and was supported by the clinical description; more specific terms were avoided when the chart did not justify that level of detail. This approach follows the purpose of HPO as a standardized hierarchical vocabulary for accurate, computable representation of patient abnormalities [30,31].

A symptom was coded as present if it had occurred at least once during the documented clinical history, even if it later remitted. Intellectual disability and its severity were based on the most recent available standardized cognitive assessment. For neurological and psychiatric symptoms, onset was defined as the age at which the first evident symptom in that domain was described. When the age at onset was not available, we used the age at the first clinical evaluation that reported or described the symptom.

For clustering, symptoms were organized into three domains. The developmental domain followed the NDD constructs of DSM-5 and of the International Classification of Diseases, Eleventh Revision (ICD-11); the psychiatric domain covered behavioral and emotional symptoms; the neurological domain covered seizures, tone, movement, coordination and pyramidal-system findings. The division serves the analysis and does not imply clear boundaries between symptoms: the three domains overlap extensively, both in clinical presentation and in genetic cause [1,2,3,4].

### 2.3. Latent Class Analysis and Profile Characterization

Clinical profiles were derived by latent class analysis (LCA), which assigns each patient a probability of belonging to each of a small number of hidden classes based on their pattern of binary indicators [12,13,33]; the approach and its reporting followed published recommendations for applied LCA [14,15,34]. Twenty-six candidate binary indicators were considered: 13 NDD, six psychiatric, and seven neurological features. The complete list, including HPO identifiers, domain assignment, prevalence, and retention or exclusion status, is provided in Table S1. Conditional symptom probabilities for the 23 retained indicators, that is, the estimated probability that a patient in a given class presents each feature, are reported in Table S4. Indicators with prevalence below 5% in the common estimation sample were excluded before fitting, because such rare features make model estimates unstable. All 128 patients had complete data for the retained indicators and entered model estimation.

One- through six-class Bernoulli LCA models were fitted by expectation–maximization [35] under conditional local independence, using multiple random starts with replication of the best log-likelihood (seed = 20260901; other estimation settings are given in the accompanying statistical report). Models were compared on log-likelihood, Akaike information criterion (AIC), Bayesian information criterion (BIC), sample-size-adjusted BIC (saBIC), class size, entropy R^2^ (a 0–1 summary of how sharply the classes separate) [36], mean maximum posterior probability, and adjacent bootstrap likelihood-ratio tests (BLRTs) for 3 vs. 4 and 4 vs. 5 classes (199 replicates each) [37]. AIC, BIC and saBIC balance how well a model fits the data against its complexity, each penalizing additional classes to a different degree, and may therefore favor different numbers of classes [14,15,34].

K denotes the number of latent classes, that is, the number of clinical profiles the model is asked to identify. BIC, the most stringent criterion, favored K = 2, whereas AIC and saBIC kept improving up to K = 6. The choice therefore rested on class size and clinical interpretability: K = 2 merged clinically distinct presentations into a single broad class, whereas K = 5 and K = 6 each produced a five-patient modal class, too small for group-level analysis. K = 4 was the smallest solution yielding classes of usable size with coherent, clinically recognizable symptom profiles, and is presented as a working solution rather than a uniquely optimal model.

We verified that the data could support reliable estimation of the model’s parameters (practical identifiability, assessed from the rank and condition number of the observed information matrix) and that the indicators were not correlated within each class (local independence, assessed from bivariate residuals) [14,15]. To test whether patients remained in the same classes when the sample changed slightly (assignment stability), the model was refitted on 250 bootstrap resamples of the original cohort, and each solution was compared with the original classification using the adjusted Rand index (1 = identical; ≈0 = chance) [38]. Each patient was assigned to the class with the largest posterior probability (modal assignment); the HC80 subset (Section 2.1) comprises the patients assigned with a probability of at least 0.80, and the model was not re-estimated after that restriction. Posterior-probability weighting was retained as a descriptive sensitivity analysis (Table S15). A formal Bolck–Croon–Hagenaars (BCH) three-step sensitivity analysis was applied to the primary profile burden comparisons (Section 2.10): in this approach, classes are estimated first, and the subsequent analysis of external variables is weighted by the estimated classification-error matrix, so that associations are not biased by assignment uncertainty [39,40,41].

Classes were labeled from conditional symptom probabilities alone, without reference to diagnoses or genetic results, so that the subsequent comparisons with both remained independent.

Profiles were then compared on a prespecified set of demographics, developmental, clinical, treatment, diagnostic, and genetic-status variables, using chi-square tests for categorical and Kruskal–Wallis tests for continuous variables. Benjamini–Hochberg correction [42], which controls the false discovery rate (FDR) and yields adjusted *p* values (q values), was applied separately within two families of tests: the 19 categorical and the 11 continuous comparisons. The variable list and the handling of sparse tables and rare events are given in the accompanying statistical report.

### 2.4. Whole-Exome Sequencing and Variant Interpretation

Genomic DNA was extracted from peripheral blood from probands and, when available, their parents. WES libraries were prepared with the Twist Exome 2.0 capture system (Twist Bioscience, South San Francisco, CA, USA) and sequenced as 150-bp paired-end reads on a NovaSeq 6000 instrument (Illumina, San Diego, CA, USA). Reads were aligned to GRCh37/hg19 with the Burrows–Wheeler Aligner [43], and single-nucleotide variants and small insertions/deletions were called with Genome Analysis Toolkit (GATK) v4.0 HaplotypeCaller [44]. Exome data were reviewed using enGenome eVAI v3.1 and Integrative Genomics Viewer [45,46]. Candidate variants were evaluated using population frequency, computational predictions, functional evidence, phenotype concordance, and segregation; classification followed American College of Medical Genetics and Genomics/Association for Molecular Pathology (ACMG/AMP) guidelines [23]. A WES finding was considered clinically informative when a pathogenic or likely pathogenic variant was judged explanatory by multidisciplinary review. On this basis, each patient was assigned an operational WES status used throughout this study: status 1, a clinically informative pathogenic or likely pathogenic finding; status 0, a negative exome; and status 2, a pathogenic or likely pathogenic variant whose causal role in the phenotype had not yet been established at the time of analysis.

### 2.5. VUS Dataset Preparation

VUS files were available for 113 of the 128 patients in the database. Variants were filtered using the following criteria: Genome Aggregation Database (gnomAD) v4.1 [47] allele frequency <1%, read depth ≥20×, at least 8 reads supporting the alternative allele, and variant allele frequency ≥35%; coding and predicted splice-altering variants were retained. For molecular analyses, VUS were defined strictly as variants classified as Uncertain significance in the source files; variants classified as pathogenic, likely pathogenic, mixed or other were excluded from VUS calculations. Within each patient, duplicate records were merged when they shared the same gene, Human Genome Variation Society (HGVS) [48] coding description, HGVS protein description, and predicted effect. Because aggregation of rare VUS does not establish pathogenicity and burden estimates can be influenced by mutation opportunity, gene constraint, ancestry, and technical heterogeneity, all pathway-level VUS analyses were treated as hypothesis-generating [23,24,47,49,50].

We built an external gene-level reference that combined coding-sequence (CDS) lengths from Matched Annotation from NCBI and EMBL-EBI (MANE) Select v1.5 [51] with consequence-specific mutation probabilities from Samocha et al. [50]. This reference was matched to the gene-level VUS counts of each tested pathway through HUGO Gene Nomenclature Committee (HGNC) identifiers rather than genomic coordinates, so that patient variants did not need to be remapped. The two annotation sets were kept separate and were not included in the burden models. The reference was used descriptively. Longer and more mutable genes accumulate more variants by chance, so we checked whether each gene’s contribution to the principal profile signal followed its coding length or expected mutability. Coverage and full contents are given in Table S22 and the accompanying statistical report.

### 2.6. Anchor Genes, Reactome Mapping, and Pathway Convergence

Anchor gene sets were defined separately within each of the four LCA profiles and each of the six non-mutually exclusive diagnostic groups. Pathway definitions were obtained from the Reactome Knowledgebase [32] using release v97 (announced 23 June 2026; gene sets downloaded 30 June 2026) [52].

Each mapped anchor gene was assigned to every Reactome pathway containing it, at all levels of the hierarchy. Because the hierarchy nests specific processes inside progressively more general ones, pathways with more than 1000 genes or at the top two hierarchy levels (levels 0–1, such as Signal Transduction, 2622 genes) were labeled as broad. Broad pathways are too unspecific to support a biological interpretation on their own. They were nevertheless retained in every analysis and in every correction family; the label only flags results that should not be interpreted as specific. Anchor genes not present in Reactome were reported but were not assigned to any pathway.

A pathway was defined as convergent when it contained at least two distinct anchor genes from the same profile or diagnosis. Convergence was evaluated with the hypergeometric test [53], which compares the number of anchor genes observed in a pathway with the number expected if the same number of genes were drawn at random from the 11,984 human genes in Reactome. Correcting this test for multiple comparisons is problematic: only pathways that already contain two anchor genes can be convergent, so the set of tests to be corrected is selected by the same count that the test measures. Convergence is therefore reported descriptively, and corrected values are not interpreted; FDR-corrected values within each cohort are reported both across all pathways containing an anchor gene and across convergent pathways only (Table S8).

### 2.7. Profile-Based VUS Analysis

The primary VUS analysis compared each LCA profile with the other three combined, among the 113 patients with a VUS file. To avoid circularity, for every tested pathway we excluded patients whose clinically informative explanatory gene was itself a member of that pathway, regardless of profile membership. This removed between zero and six patients per pathway (zero when the patients carrying the anchor gene had no VUS file), leaving 107–113 patients per test. This design is referred to as the pathway-specific primary analysis.

For each pathway, the outcome was the number of filtered VUS a patient carried in any of its member genes. This count was modeled by Poisson regression with the patient’s total VUS count as an offset, so that groups were compared on the rate at which variants fall in the pathway rather than on raw counts. Standard errors were heteroskedasticity-consistent (HC3) [54], and the effect estimate was an incidence-rate ratio (IRR). Two secondary endpoints were also analyzed: a two-sided Mann–Whitney U test of the patient-level pathway-VUS proportion, and carrier status—at least one VUS in the pathway—tested by two-sided Fisher exact test and by logistic regression adjusted for standardized total VUS with HC3 standard errors. Carrier models affected by complete or quasi-complete separation do not allow valid inference and are reported as unavailable; where every eligible patient was a carrier, the Fisher test returns *p* = 1 and the comparison is reported as uninformative. The count-based endpoints are not affected by these issues.

### 2.8. Diagnosis-Based VUS Analysis

The complete analysis was repeated for ASD, ADHD, SLD, the speech-language disorder grouping (Sp-Lan-D), DCD, and ID. Diagnoses were non-mutually exclusive; for each diagnosis, cases were patients with that diagnosis and controls were patients without it, who could have other NDD diagnoses. Diagnosis-specific clinically informative WES genes defined the pathway universe, and the same VUS outcomes, offset, estimators, confidence intervals, and multiplicity rules were used.

### 2.9. Comparison of Profile- and Diagnosis-Based Analyses

Each clinical profile was paired with one diagnosis based on its prevalence within the profile, its enrichment relative to the other profiles, and clinical fit; where these criteria diverged, specificity and enrichment were prioritized over raw prevalence. Profile–diagnosis association was described by the number of patients carrying both labels, by the Jaccard index—the overlap divided by the number of patients carrying either label, equal to 1 for identical groups and to 0 when the two labels never co-occur [55]—and by a one-sided Fisher exact test of whether the diagnosis was more frequent within the profile than outside it.

A pathway counted as a positive-direction association for a group when, in the pathway-specific primary analysis, its incidence-rate ratio exceeded 1—that is, patients in that group carried more VUS in the pathway than the comparator patients—with a two-sided *p* < 0.05 before correction for multiple testing (a nominal result). Such pathways are therefore candidate hypotheses, rather than findings established after FDR correction. A result in the opposite direction—an incidence-rate ratio below 1, that is, fewer pathway VUS in the group than in the comparator—is termed an inverse finding. Inverse findings are reported for completeness but are not counted among the positive-direction associations, because a relative depletion of uncertain variants may arise from biological or technical factors, such as differences in coverage or annotation. For a given Reactome pathway, profile- and diagnosis-based tests used the same pathway-specific eligible patient set, allowing direct comparison of effect direction without introducing different circularity exclusions. Profile–diagnosis patient overlap was described in the 113 patients with a VUS file. Secondary analyses that required the same patients for every pathway—testing whether pathways found in one framework carried over to the other, joint models, and repeated subsampling with groups of equal size in the two frameworks—used the global informative-WES exclusion cohort (Section 2.10) and are reported as sensitivity analyses. The one exception is the head-to-head comparison of profile C3 with diagnosis ADHD on their shared pathway (Section 3.4), whose joint models are fitted on the pathway-specific primary cohorts (*n* = 111, and *n* = 99 in HC80).

### 2.10. HC80 and Additional Sensitivity Analyses

HC80 comprised 114 of the 128 patients, 101 of whom had a linked VUS file. The profile and diagnosis analyses were repeated within it, with anchor sets redefined from its clinically informative WES cases and the same pathway-specific exclusion rule applied, leaving 96–101 eligible patients per test.

Four additional cohorts tested the same signals under stricter or looser eligibility criteria. The global informative-WES exclusion cohort removed every status 1 patient with a VUS file, irrespective of pathway, so that no result depended on patients with a genetic diagnosis. The strict WES-negative cohort also removed the unresolved status 2 patients, leaving only patients with a negative exome. The unfiltered cohort of all 113 patients with a VUS file applied no exclusions and served only to show the effect of the exclusions. To prevent a few patients from driving the results, the outlier-trimmed cohort excluded patients with an unusually high number of VUS, defined as those above the Tukey upper fence for total filtered VUS, that is, the third quartile plus 1.5 times the interquartile range (IQR) [56]; because it does not control gene-to-pathway circularity, it is reported as a robustness check rather than as an eligibility rule. The principal profile signal was further examined using sex- and age-adjusted Poisson models, posterior-probability weighting, and leave-one-observed-gene-out and leave-one-anchor-out analyses. Cohort sizes and composition are given in the accompanying statistical report.

The BCH sensitivity analysis was applied to all 224 primary profile–pathway comparisons, using the same offset and eligibility rule as the primary models. Inference was evaluated both conditional on the fitted classification-error matrix and after 1000 nonparametric bootstrap refits of the K = 4 LCA [57]; implementation and diagnostics are reported in Table S21 and statistical-report worksheets 55–57.

### 2.11. Multiplicity, Direction of Reporting, and Reproducibility

FDR correction was applied within families defined by analysis cohort and endpoint. Broad pathways remained in their families; models reported as unavailable (Section 2.7) have no p or q value and were not included. The BCH sensitivity analysis is the exception: its conditional and LCA-refit bootstrap results form separate families that retain all 224 hypotheses, with unavailable tests entered as *p* = 1, the most conservative value (Table S21). Nominal primary and HC80 findings are given in Tables S10–S12; the complete set of tests, including all endpoints and nonsignificant results, is provided in the accompanying statistical report, which also documents software versions, frozen file hashes and the quality-control checks (Table S20). Analyses used Python 3.12, with the Poisson and logistic estimators and their HC3 covariance implemented directly in the analysis code.

## 3. Results

### 3.1. Clinical Profiles

**Figure 2:**
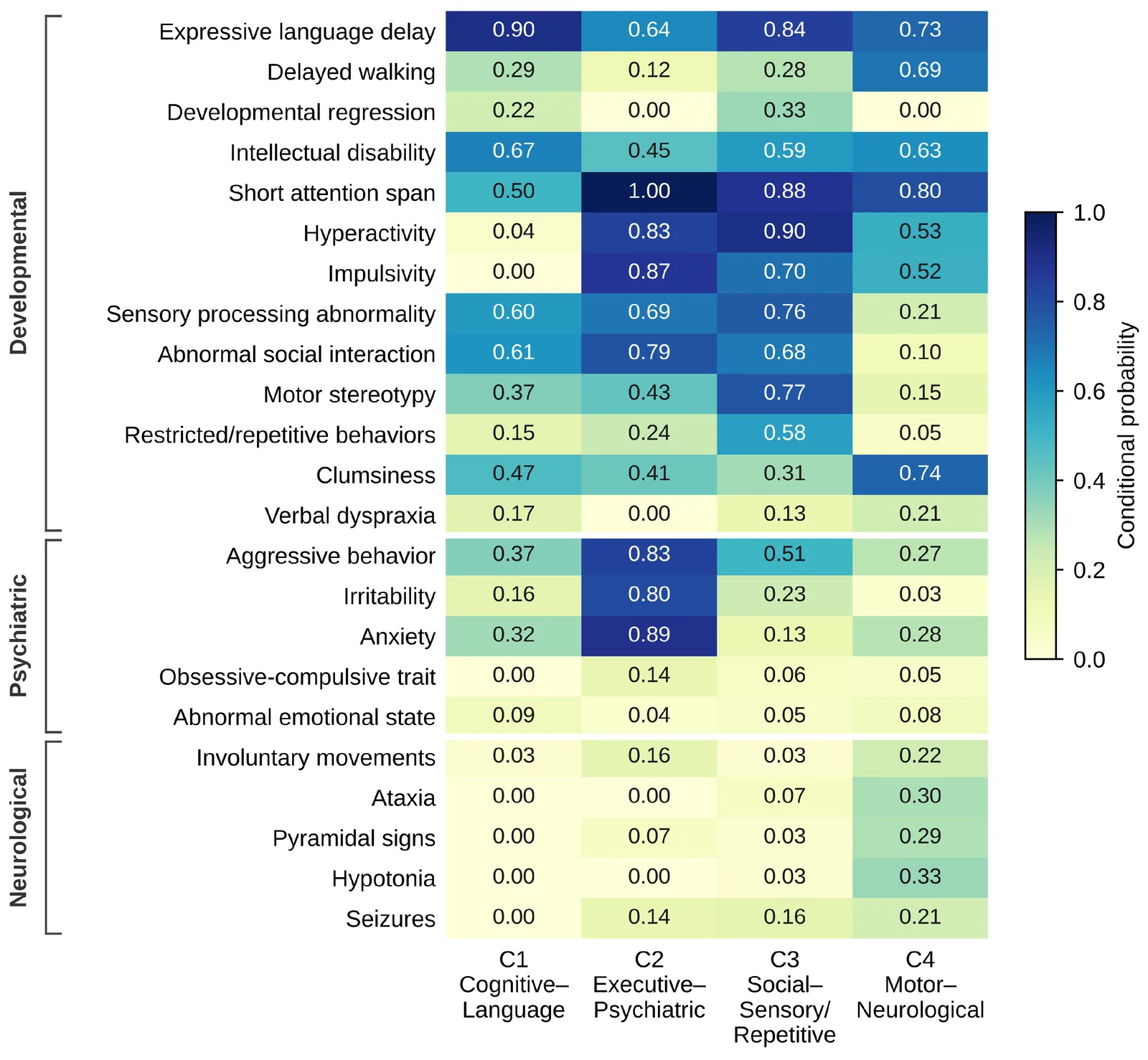
Conditional symptom probabilities for the four clinical profiles. Each cell shows the estimated probability that a patient in the profile presents the feature, from 0 (light) to 1 (dark); features are grouped by domain.

Modal assignment produced four profiles, referred to as C1–C4 throughout: C1 Cognitive–Language (*n* = 34), C2 Executive–Psychiatric (*n* = 30), C3 Social–Sensory/Repetitive (*n* = 41), and C4 Motor–Neurological (*n* = 23), of whom HC80 retained 29, 25, 39, and 21 patients, respectively (114/128 overall, 89.1%). Each profile is described by its conditional symptom probabilities, shown in full in Figure 2 and tabulated in Table S4.

C1 was dominated by expressive-language delay, with intellectual disability in about two-thirds of its patients and almost no hyperactivity or impulsivity. C2 combined a short attention span, present in nearly every patient, with hyperactivity, impulsivity, aggression, irritability, and anxiety. C3 combined sensory-processing abnormality, abnormal social interaction, and stereotypy, with restricted and repetitive behaviors in about half of its patients and regression in a minority. C4 was defined by delayed walking and clumsiness, with less frequent ataxia, pyramidal signs, and hypotonia, and low probabilities of social, sensory, and repetitive features.

The profiles were then compared on demographic, developmental, treatment and neurodiagnostic variables that were not included in the latent class model, in order to describe them on external features. Two clinical variables differed across profiles after FDR correction. Age at independent walking was later in C4 (median 19 months) than in the other three profiles (14–14.5 months; *p* = 0.00051, q = 0.0056), and pharmacological treatment was most frequent in C2 (76.7%) and least frequent in C1 (20.6%), with intermediate rates in C3 and C4 (53.7% and 43.5%; *p* = 0.00012, q = 0.00073). Sex, age at clinical evaluation, age at speech acquisition, intellectual-disability severity, and EEG or brain-imaging abnormalities did not differ (all q > 0.05). Table 1 reports these comparisons for sex, age at evaluation, age at walking and pharmacological treatment; the complete set is given in the statistical report. The correspondence between profiles and clinical diagnoses is examined in Section 3.4.

The four classes were sharply separated (entropy R^2^ 0.866), with no evidence against local independence. They were, however, only moderately stable when the model was refitted on resampled data (median adjusted Rand index 0.315, IQR 0.239–0.394), and 42.4% of refits produced a class of fewer than 10 patients. This pattern is consistent with an exploratory clinical representation rather than a stable taxonomy. Model-selection indices for K = 1–6 and full model diagnostics are given in Tables S2 and S3.

### 3.2. Profile Anchor Pathways and Convergence

The 26 patients with a clinically informative WES finding contributed 27 profile-level anchor-gene occurrences; one patient contributed two genes (WES in P_034 was informative for both *SMPD4* and *TRRAP*). Anchor-set sizes, successfully mapped genes, and the resulting pathway universes are given by profile in Table 2 and Table S7. Convergence—at least two within-profile anchor genes in the same pathway—was almost entirely confined to C3, which accounted for 23 of the 28 convergent profile–pathway pairs, with four in C4, one in C2 and none in C1; eight of the 28 were not broad. Corrected significance is not interpreted, because the correction family is defined by the anchor count that drives the test (Table S8).

The most specific convergent pathways in C3 included Interaction between L1 and Ankyrins (*L1CAM* and *SCN2A*; 31 genes; hierarchy level 4; *p* = 0.00029), Neurexins and neuroligins (*NLGN1* and *SHANK3*; 57 genes; hierarchy level 2; *p* = 0.00098), *L1CAM* interactions (*L1CAM* and *SCN2A*; 121 genes; hierarchy level 3; *p* = 0.0043), and Axon guidance (*L1CAM*, *SCN2A*, and *SHANK3*; 542 genes; hierarchy level 2; *p* = 0.0087). C2 convergence involved the broad Chromatin organization pathway through *ADNP* and *KMT2C*. C4 convergence was mainly confined to broad transcriptional and signal-transduction pathways. These anchor-convergence tests describe co-membership of known WES genes; they are distinct from VUS enrichment tests.

### 3.3. Profile-Based VUS Enrichment

The 113 VUS files contributed 59,950 filtered VUS after deduplication. In the pathway-specific primary analysis the eligible sample varied from 107 to 113 patients across the 224 profile–pathway tests. Across these primary tests, one positive and three inverse Poisson associations were nominal; none survived FDR correction.

**Figure 3:**
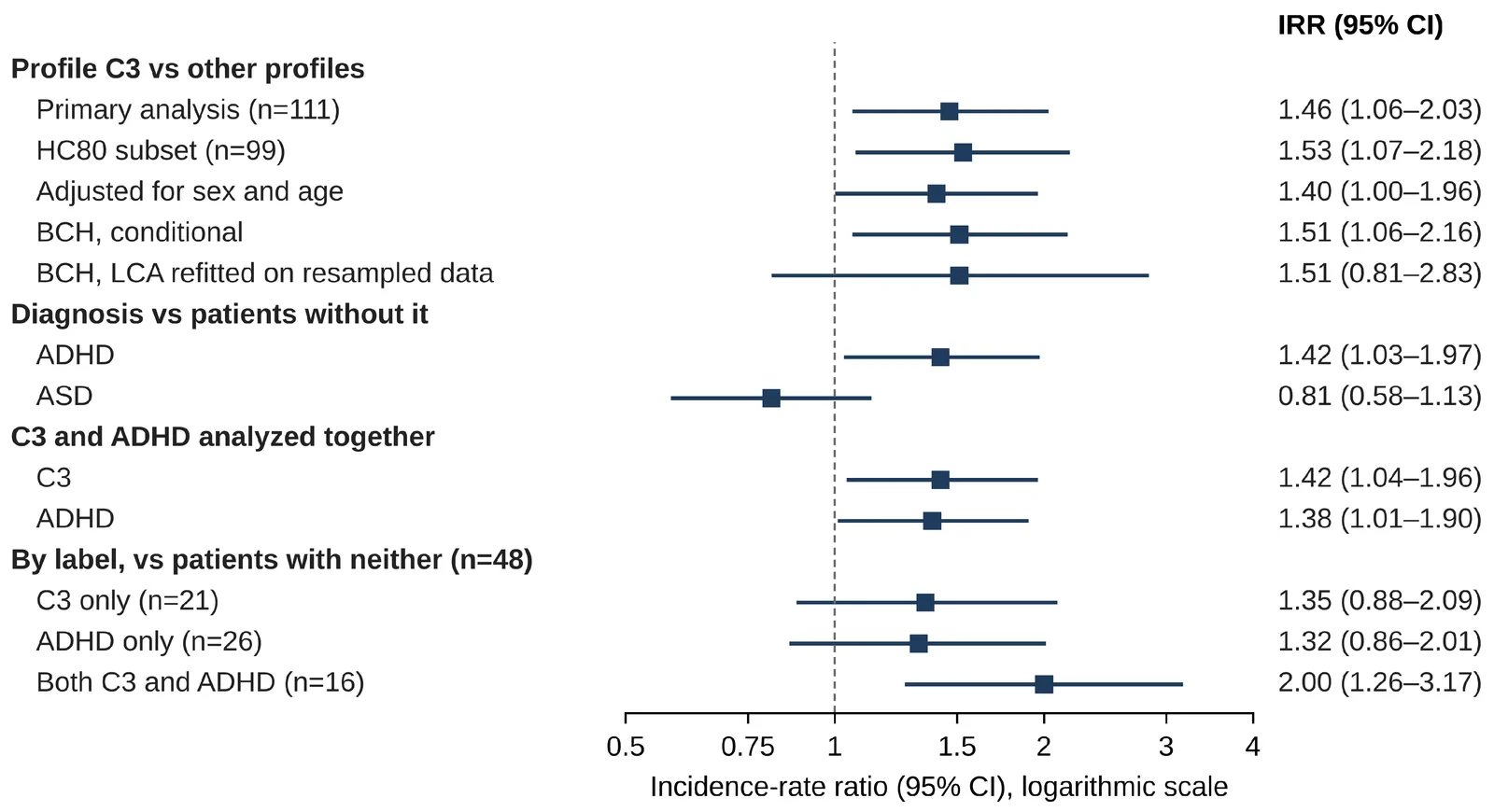
Burden of variants of uncertain significance (VUS) in Transcriptional Regulation by *MECP2* across groups and analyses. Each row shows an incidence-rate ratio (IRR) with its 95% confidence interval (CI); values above 1, to the right of the dashed line, indicate more VUS in the pathway in that group than in the comparison patients. The axis is logarithmic. The primary analysis compares profile C3 with the other three profiles in the 111 eligible patients; the other rows show the checks described in Section 3.4 and Section 3.5. BCH, Bolck–Croon–Hagenaars correction for classification error; LCA, latent class analysis; HC80, high-confidence subset.

The only positive-direction finding in the primary profile analysis was Transcriptional Regulation by *MECP2* in C3 (63 genes; hierarchy level 3). The excess was spread across the gene set rather than concentrated in *MECP2* itself, so the pathway name does not imply an *MECP2*-specific mechanism. The only two patients in the cohort whose clinically informative finding lay in this pathway, carrying *RBFOX1* and *TBL1XR1*, both had a VUS file and were therefore excluded, leaving 111 patients (37 C3 and 74 other profiles). Mean pathway-VUS counts were 1.65 versus 1.12; the offset model yielded an IRR of 1.46 (95% CI 1.06–2.03; *p* = 0.0223; q = 0.921; Figure 3). Carrier frequencies were 29/37 (78.4%) versus 50/74 (67.6%; Fisher *p* = 0.273; adjusted logistic *p* = 0.269); thus, the carrier endpoints were not nominally significant.

The three inverse primary profile findings were Signaling by Hedgehog in C1 (IRR 0.68; *p* = 0.00919) and two taste-perception pathways in C3 (IRR 0.62 and 0.65; *p* = 0.0164 and 0.0471), all with q = 0.921. All nominal profile associations in both directions, including HC80 findings, are listed in Table S10; positive and inverse diagnosis findings are listed in Tables S11 and S12. Adjusted carrier logistic models could not be estimated because of separation in 30 profile and 98 diagnosis comparisons, and no carrier logistic comparison was FDR-significant in any framework or cohort (Table S9).

### 3.4. Diagnosis-Based VUS Enrichment and Framework Comparison

In the 128-patient clinical cohort, the number of patients with each diagnosis was ASD 80, ADHD 46, SLD 7, Sp-Lan-D 90, DCD 12, and ID 59. Because primary exclusions were pathway-specific, diagnosis case and control counts varied across the 603 group–pathway tests; the total eligible sample size ranged from 107 to 113. Twenty-one positive-direction and 11 inverse Poisson associations were nominal, but none survived FDR correction.

The strongest positive diagnosis signal was Metabolism in ADHD, a broad pathway of 2197 genes at the top of the Reactome hierarchy. Three informative-WES patients with a VUS file were excluded because their explanatory genes belonged to the pathway, leaving 110 patients (41 ADHD and 69 without ADHD); the IRR was 1.12 (95% CI 1.05–1.19; *p* = 0.000871; q = 0.353). The remaining positive nominal signals, listed with their estimates in Table S11, involved metabolic, transport, and transcriptional pathways across ADHD, SLD, and ID; 10 of the 21 were broad. Because the Reactome hierarchy nests pathways within one another, the number of nominal pathways overstates the number of independent biological signals.

The inverse diagnosis findings were concentrated in ID, where *HOX*-gene and chromatin-related pathways showed lower VUS rates (IRR 0.53–0.79; p from 0.00180 to 0.0164; Table S12); none survived FDR correction. Because these directions are counterintuitive, we checked whether they reflected a global difference in variant burden: in all 113 patients with a VUS file, total VUS counts did not differ detectably between patients with and without a DSM-5 ID diagnosis (median 509 in 50 patients with ID versus 511 in 63 without; two-sided Mann–Whitney *p* = 0.85). This argues against a global explanation, although it does not rule out pathway-specific confounding or sampling variability.

C1 paired with Sp-Lan-D, C2 with ADHD, C3 with ASD, and C4 with DCD. In the 113 patients with a VUS file, overlap/Jaccard values were 23/0.267, 19/0.380, 30/0.390, and 6/0.250, respectively (Table S13). The two frameworks shared one positive-direction association: Transcriptional Regulation by *MECP2*, which was nominal in C3 and also in ADHD, rather than in ASD, the diagnosis paired with C3. The secondary analyses described in Section 2.9, which tested whether pathways carried over from one framework to the other and compared groups of equal size, did not show an advantage for the profiles and were treated as sensitivity analyses.

Because this was the only shared nominal association, and because the two groups in which it emerged were not paired with each other, profile C3 and ADHD were compared head to head on the same patients with the same offset model (Table S23; Figure 3). The two labels largely identified different patients, overlapping in 16 of the 63 patients carrying either label (Jaccard 0.25; Fisher *p* = 0.42). When both were entered in the same model, C3 and ADHD each remained associated with the pathway (C3 IRR 1.42, *p* = 0.030; ADHD IRR 1.38, *p* = 0.044), with no evidence that one was stronger than the other (ratio 1.03, 95% CI 0.66–1.59). Patients carrying both labels had the highest burden (IRR 2.00, 95% CI 1.26–3.17); the interaction was not significant (*p* = 0.74). Two analyses qualified this result: in HC80 the ADHD estimate was no longer nominal (*p* = 0.083), and both estimates weakened after adjustment for sex and age (C3 *p* = 0.053; ADHD *p* = 0.063).

ASD, the diagnosis paired with C3, showed an inverse association with the pathway. On its own, its estimate was below 1 but not nominally significant (IRR 0.81; *p* = 0.21); it became nominal once C3 was included in the same model (IRR 0.71, 95% CI 0.52–0.98; *p* = 0.040), while the C3 estimate rose to 1.61 (*p* = 0.0049).

### 3.5. HC80 and Robustness Analyses

Within the 101 HC80 patients with a VUS file, pathway-specific exclusion left 96–101 eligible patients per test; the 12 patients with a VUS file removed by this restriction (out of the 14 excluded from the clinical cohort) are characterized in Table S14. Four positive and three inverse Poisson associations were nominal, and none survived FDR correction. The C3 association persisted (IRR 1.53; *p* = 0.0188). Three further positive associations appeared only under the HC80 restriction—Signal Transduction and Signaling by GPCR (G protein-coupled receptors) in C1, and Myogenesis in C4—and none had a counterpart in the diagnosis-based HC80 analysis (Table S10). In the two C1 pathways, every eligible patient was a carrier, so these associations reflect relative burden rather than carrier enrichment. The diagnosis-based HC80 analysis produced 13 positive and nine inverse nominal associations, again with none FDR-significant; Hemostasis in ADHD was the only one not already nominal in the full analysis.

In the global informative-WES exclusion and strict WES-negative cohorts, the samples were smaller (93 and 84 patients, respectively). In both, the only FDR-significant results were in the diagnosis framework, and all concerned signals already nominal in the primary analysis: higher metabolic-pathway burden in ADHD (Metabolism, q = 0.026–0.040; Metabolism of lipids, q = 0.041) and lower chromatin-pathway burden in ID (q = 0.023–0.041). The C3 association kept its direction in both, as it did in the outlier-trimmed cohort, whereas Myogenesis strengthened under the stricter rules. The outlier-trimmed cohort produced the only FDR-significant profile result in any cohort: an inverse association with Signaling by Hedgehog in C1 (IRR 0.57; q = 0.042). Because that cohort does not control circularity, this is a robustness observation rather than evidence of protective biology. Conclusions therefore depend on how the cohort is defined (statistical-report worksheets 24, 25, 30 and 31).

The principal profile signal proved stable in direction but fragile in significance. The C3 estimate remained positive after adjustment for sex and age (IRR 1.40; *p* = 0.050), after formal BCH correction for classification error (IRR 1.51) and after removal of each of the 42 observed genes in turn, but it lost nominal significance under sex/age adjustment, in three of the 42 gene removals and after bootstrap refitting of the LCA (Figure 3; Tables S16, S18 and S21). Within the pathway, gene-level VUS counts tracked coding length and expected mutability (Spearman ρ = 0.50), whereas the C3 excess tracked neither (ρ = −0.06 and −0.04); with only 42 genes, however, this does not rule out confounding by mutation opportunity (Table S22).

## 4. Discussion

Transdiagnostic work has shown that neurodevelopmental categories are internally heterogeneous and mutually overlapping, and that data-driven profiles capture combinations of developmental, behavioral and neurological features that cut across diagnostic boundaries [3,4,5,6,7,8,9]. Large sequencing studies have shown that the genes implicated in NDDs converge on a limited number of biological processes [16,17], while routine exome testing leaves many families with variants of uncertain significance that cannot support clinical decisions [18,20,23,24,25]. These two lines of research are rarely combined, and when they are, the direction is usually from genes to phenotypes: Shimelis et al. clustered 263 NDD genes by the phenotype frequencies of about 9000 probands and found that the resulting clusters enrich distinct biological processes [58]. Phenotype-first studies work in the opposite direction but stop at different points [29,59]. Litman et al. decomposed autism phenotypes into classes with distinct genetic programs in thousands of individuals [27], Warrier et al. related phenotypic heterogeneity to genetic correlates [26], and cluster-based clinical work describes profiles without genomic follow-up [28]. A recent review of data-driven subtyping in autism lists methodological inconsistency and the integration of categorical and dimensional models among the open problems of the field [60]. What remains untested is whether, in the same patients, a data-driven grouping and the clinical diagnoses point to different biology, and whether the variants left unresolved by routine testing can support that comparison.

This study adopts a phenotype-first approach. Candidate pathways are defined by clinically informative WES findings, to avoid circularity, no patient contributes to the tests of pathways containing their own explanatory gene. The profile- and diagnosis-based analyses then run on the identical eligible sample, which is what makes the two groupings comparable. The outcome is the residual VUS burden, that is, the part of the exome data left unused by routine testing.

Grouping the same patients in two different ways highlighted partly different pathways. The diagnosis framework returned more signals, but it also tested nearly three times as many hypotheses in overlapping groups. The two met on one pathway, Transcriptional Regulation by *MECP2*. The groups in which it emerged, C3 and ADHD, were not paired by design, and they largely identify different patients. Two interpretations are possible: the pathway may be involved in more than one clinical presentation, or it may relate to a feature shared by part of C3 and part of ADHD that neither grouping isolates on its own. This cohort cannot distinguish between them.

Restricting the analysis to the least ambiguous profile assignments changed the results: three pathways that had not reached *p* < 0.05 in the full cohort did so in HC80. Clearer class assignment may sharpen real differences between groups easier to detect, or the smaller sample may simply shift borderline results; these data cannot distinguish between the two explanations. The restriction did reveal candidate pathways that the diagnosis-based analysis did not, including Myogenesis in C4. With 128 patients and no result surviving FDR correction, these remain hypotheses. It would be worthwhile to apply the same approach to larger cohorts, where classes separate more sharply and results can withstand correction for multiple testing, and validating the profiles themselves independently [11].

### Limitations

Several limitations constrain these conclusions. The study is single-center and exploratory, with 128 patients, classes of 23 to 41, 15 patients without a VUS file, and no independent validation cohort. No result survived FDR correction in the primary analysis, and corrected significance changed with cohort definition, so no finding is confirmatory; at this sample size, only large effects were detectable, as shown by the minimum detectable effects at approximately 80% power (Table S19). The data lack genotype-derived ancestry components, sequencing depth, callable sequence, batch and year, so residual confounding by technical factors or ancestry cannot be excluded; gene-level counts are not normalized for coding length or gene-specific mutability, and the external annotations used descriptively cannot substitute for patient-level callable sequence. Symptoms were recorded only as present or absent over the whole clinical history, without information on severity, frequency, duration or functional impact, and were abstracted from records spanning a long period, with different instruments and assessors. The K = 4 solution is a working choice rather than a uniquely supported one: the 4→5 BLRT was significant, bootstrap agreement was moderate, and a minority of refits produced very small classes. Anchor-defined pathway universes depend on rare index cases: each principal profile pathway was included on the basis of a single anchor gene (Table S17). Carrier endpoints are uninformative for broad pathways. VUS recurrence establishes neither pathogenicity nor mechanism, and VUS classifications change over time [61].

## Figures and Tables

**Table 1:** Clinical characteristics by K = 4 profile, restricted to the variables reported in this section: age at independent walking and pharmacological treatment, which differed after false discovery rate (FDR) correction, and sex and age at last evaluation, which did not. Profiles are C1 Cognitive–Language, C2 Executive–Psychiatric, C3 Social–Sensory/Repetitive and C4 Motor–Neurological. Continuous variables are median [interquartile range, IQR]. Categorical variables were compared by Pearson chi-square and continuous variables by Kruskal–Wallis, with Benjamini–Hochberg q values (*p* values adjusted for multiple testing) computed within the categorical (19 tests) and continuous (11 tests) families. Conditional symptom probabilities, assignment quality, and VUS sample characteristics are reported in Tables S4–S6. Complete clinical comparisons are provided in the statistical report, worksheets “13 Clinical Cat Tests”, “14 Clinical Cat Levels”, and “15 Clinical Numeric”.

Characteristic	C1 (*n* = 34)	C2 (*n* = 30)	C3 (*n* = 41)	C4 (*n* = 23)	*p* Value	q Value
Male, n (%)	18 (52.9)	23 (76.7)	27 (65.9)	14 (60.9)	0.256	0.441
Age at last evaluation, y	9.0 [7.0–11.0]	8.5 [6.0–12.0]	8.0 [6.0–11.0]	11.0 [7.0–13.0]	0.297	0.618
Age at independent walking, mo	14.5 [12.0–18.0]	14.0 [12.0–15.0]	14.0 [13.0–18.0]	19.0 [16.0–21.5]	0.00051	0.0056
Pharmacological treatment, n (%)	7 (20.6)	23 (76.7)	22 (53.7)	10 (43.5)	0.00012	0.00073

**Table 2:** Profile-specific clinically informative WES anchor sets and Reactome pathway universes derived from the full 128-patient clinical cohort. For primary VUS testing, a patient supplying a clinically informative WES gene was excluded only from pathways containing that explanatory gene; informative-WES patients remained eligible for unrelated pathways.

Class	Profile	Anchor Genes	Mapped	Anchor Pathways	Convergent	Broad >1000	Levels 0–1
C1	Cognitive–Language	*ANKRD11*; *KAT6A*; *PRKAR1B*; *SETBP1*; *TRIP12*	3	61	0	9	22
C2	Executive–Psychiatric	*ADNP*; *ATRX*; *KMT2C*	3	41	1	7	14
C3	Social–Sensory/Repetitive	*CLCN4*; *L1CAM*; *LZTR1*; *MBD5*; *MED13L*; *MYT1L*; *NLGN1*; *SCN2A*; *SHANK3*; *SMPD4*; *TBL1XR1*; *TRRAP*	10	97	23	11	39
C4	Motor–Neurological	*AUTS2*; *PHIP*; *PURA*; *RBFOX1*; *SRCAP*; *TCF4*; *TMEM240*	4	25	4	5	10

Mapped, anchor genes present in Reactome; Anchor pathways, pathways containing at least one anchor gene; Convergent, pathways containing at least two anchor genes; Broad > 1000, anchor pathways with more than 1000 genes; Levels 0–1, anchor pathways at the top two levels of the Reactome hierarchy.

## Data Availability

De-identified aggregate analysis tables, complete pathway-level results, and a privacy-reduced patient-level audit report go with this manuscript. Exact dates, original laboratory sample identifiers, patient-level HGVS variant records, and granular clinical/genetic fields with elevated re-identification risk are not included in the public Supplementary Files. The accompanying files are Supplementary_materials.docx, Statistical_Report.xlsx, Supplementary-Patient_Level_Report.xlsx, Gene_CDS_Mutability_Reference.xlsx and Reproducibility_Verification.docx. Sharing of individual-level genomic data is still subject to consent and institutional policy.

## Supplementary Materials

The following supporting information can be downloaded at: https://www.mdpi.com/article/10.3390/diagnostics16213674/s1, Supplementary_materials.docx has Tables S1–S23. Statistical_Report.xlsx has the complete pathway-level results, the method freezes and the file manifests. Supplementary-Patient_Level_Report.xlsx has the privacy-reduced patient-level audit. Gene_CDS_Mutability_Reference.xlsx has the external gene-level CDS and mutability reference. Reproducibility_Verification.docx documents the reproducibility checks. References [41,50,51,57] are cited in the Supplementary Materials.

## Abbreviations

ADHD, attention-deficit/hyperactivity disorder; ASD, autism spectrum disorder; CI, confidence interval; DSM-5, Diagnostic and Statistical Manual of Mental Disorders, Fifth Edition; FDR, false discovery rate; HC80, high-confidence subset (posterior class-assignment probability ≥0.80); HPO, human phenotype ontology; ID, intellectual disability; IRR, incidence-rate ratio; LCA, latent class analysis; NDD, neurodevelopmental disorder; SLD, specific learning disorder; Sp-Lan-D, speech-language disorder grouping; VUS, variant(s) of uncertain significance; WES, whole-exome sequencing.
