# Supplementary material for: Integrating a Transdiagnostic Clinical Approach with Whole-Exome Sequencing in Neurodevelopmental Disorders: A Single-Center Exploratory Study
Source: Diagnostics (Basel). 2026 Nov 6;16(21):3674. doi: 10.3390/diagnostics16213674 (PMC13763132; doi:10.3390/diagnostics16213674)
Supplement: Supplementary file 1 [file diagnostics-16-03674-s001.zip › Reproducibility_Verification.pdf]

Reproducibility Verification

Integrating a Transdiagnostic Clinical Approach with Whole-Exome Sequencing in Neurodevelopmental Disorders: A Single-Center Exploratory Study

1. Purpose and scope

This document reports an independent verification that the analysis presented in the manuscript can be reproduced from the primary data and that the figures given in the manuscript, the supplementary tables, the machine-readable statistical report and the patient-level report agree with one another.

The verification was carried out on the frozen study materials, without reuse of any intermediate file of the analysis pipeline: the variant-level exports were reparsed, the outcome tables were rebuilt from the declared filter definitions, and the pathway models were reimplemented and refitted from first principles. The materials examined were Statistical_Report.xlsx, Supplementary-Patient_Level_Report.xlsx, the supplementary tables, the manuscript, the source clinical database and the 113 per-patient variant exports.

2. Reconstruction of the variant pipeline

The 113 per-patient variant exports were mapped one-to-one onto the 113 patients with a VUS file using the laboratory codes recorded in the clinical database. The filters specified in the method freeze were then applied independently: classification exactly “Uncertain significance”, coverage ≥ 20, alternate reads ≥ 8, alternate fraction ≥ 0.35 and population frequency < 1%, followed by within-patient deduplication on gene, coding change, protein change and effect.

Pipeline step	Independent rebuild	Reported
Rows classified “Uncertain significance”	59,991	59,991
Rows failing the quality/frequency filters	19	19
Eligible VUS rows	59,972	59,972
Within-patient duplicate rows removed	22	22
Final strict VUS	59,950	59,950
Per-patient VUS totals, 113 patients	all agree	worksheet 05, patient report
Per-patient unique gene counts	all agree	worksheet 05, patient report
Tukey upper fence; outlying patients	613; 7	613; 7

Every declared quantity of the variant pipeline is therefore recovered exactly, including the number of rows removed at each step. The frequency filter was applied by the variant-annotation platform (enGenome eVAI v3.1, gnomAD v4.1 population frequencies); the exports carry the platform version but not the gnomAD release, which the reporting laboratory has confirmed as v4.1 and which is recorded in the method freeze and in Table S20. Per-patient coverage metrics, callable sequence and sequencing batch were not available in the frozen exports, as the manuscript states among its limitations.

3. Reconstruction of the pathway models

The primary burden model — Poisson regression with a log link, the group indicator as predictor, log(total filtered VUS) as offset and HC3 heteroskedasticity-consistent covariance — was reimplemented directly, as in the frozen pipeline, and refitted for the C3 / Transcriptional Regulation by MECP2 comparison on four cohorts.

Cohort	n	Independent refit, IRR (p)	Reported, IRR (p)
Pathway-specific primary, ALL	111	1.462165 (0.022251)	1.462165 (0.022251)
Pathway-specific primary, HC80	99	1.529831 (0.018811)	1.529831 (0.018811)
Unfiltered audit, ALL	113	1.445101 (0.023451)	1.445101 (0.023451)
Unfiltered audit, HC80	101	1.505133 (0.020622)	1.505133 (0.020622)

Group sizes, carrier counts and event totals agree in every cohort (61/83, 58/69, 63/86 and 60/72 pathway events for cases and controls respectively), as do the confidence intervals. The pathway-specific circularity rule was also reproduced independently: starting from the 113 patients with a VUS file, exactly two carriers of a clinically informative finding in a member gene of the tested pathway (RBFOX1 and TBL1XR1) are removed, leaving the 111 patients of the primary test.

The refit was then extended from the principal signal to the whole primary analysis. Pathway membership was taken from the frozen catalog, the circularity exclusion was recomputed pathway by pathway, and the model was refitted for every pathway-specific primary comparison of both cohorts.

Family refitted independently	Comparisons	Agreement with the workbook
Profile, ALL and HC80	224 + 190	Group sizes identical; IRR and p agree to better than 1×10^{-8}
Diagnosis, ALL and HC80	603 + 570	Group sizes identical; IRR and p agree to better than 1×10^{-8}

All 1,587 primary comparisons therefore reproduce from the raw exports, not only the reported signals. The same refit supplies the sex- and age-adjusted and full (sex, age, trio) models on these cohorts, which are recorded in worksheet 61; the two rows quoted in the manuscript and in Table S16 are reproduced exactly (C3 / Transcriptional Regulation by MECP2, IRR 1.399323, 95% CI 0.999981–1.958143, p = 0.050013; ADHD / Metabolism, IRR 1.057493, p = 0.084772). No adjusted comparison reaches FDR significance in either framework or cohort.

4. Leave-one-observed-gene-out

The robustness analysis for the principal profile signal was refitted gene by gene on both cohorts. On the pathway-specific primary cohort the analysis tests 42 observed genes, preserves the positive direction after all 42 removals and nominal significance after 39, as reported in Supplementary Table S18 and in worksheet 59; nominal significance is not retained after removing HIPK2 (p = 0.0507), NCOR2 (p = 0.0537) or TNRC6C (p = 0.0720). On the unfiltered cohort of worksheets 50 and 51 the analysis tests 43 genes, with 43 of 43 and 40 of 43 respectively. The one-gene difference between the two cohorts is accounted for: BDNF carries its only pathway events in one of the two patients removed by the circularity rule and therefore has no events in the primary cohort.

5. Anchor convergence and its correction family

A pathway enters the anchor-convergence test only when it already contains at least two anchor genes of the group, and the hypergeometric statistic is driven by that same count. Correcting within the selected set therefore conditions on the selection. Both families were recomputed independently from the anchor tables and the Reactome universe, and both are carried in the workbook so that the effect of the conditioning is visible.

Family	Cohort	Selected rows	q<0.05, convergence family	q<0.05, anchor-pathway family
Profile (worksheets 22, 23)	ALL	28 of 224	15	5
Profile (worksheets 22, 23)	HC80	27 of 190	14	11
Diagnosis (worksheets 28, 29)	ALL	87 of 603	42	20
Diagnosis (worksheets 28, 29)	HC80	64 of 570	25	12

The hypergeometric p values were recomputed for every anchor pathway, not only for the convergent ones, and reproduce the reported values exactly where these were present. For the pathway quoted in the manuscript — C3 / Axon guidance — the test gives p = 0.008699, q = 0.0221 within the convergence family and q = 0.0571 within the anchor-pathway family. The manuscript reports the uncorrected p values for the convergent pathways it names and does not interpret their corrected significance; anchor convergence is descriptive throughout and no inference rests on it.

6. Non-estimable models and multiplicity

Some comparisons cannot support inference and are reported as non-estimable rather than as estimates. The verification confirms the diagnosis in each case.

Carrier logistic models. Thirty profile and 98 diagnosis comparisons are reported as unavailable because of complete or quasi-complete separation. Each was confirmed to carry a zero cell in the 2×2 carrier table or a divergent coefficient; representative cases have all cases carrying a variant (for example 37 of 37 against 74 of 75 controls) with an accompanying Fisher exact p of 1.00. After their exclusion, no estimable adjusted-logistic comparison is FDR-significant in any cohort, consistent with the Poisson, Mann–Whitney, Fisher and BCH endpoints.

Trio-adjusted Poisson models. Ten comparisons in worksheet 42 (ASD against five telomere pathways, in the ALL and HC80 cohorts) are reported as non-estimable. Each concerns a contrast carrying a single event, for which the negative-binomial counterpart is likewise non-estimable, while the sex- and age-adjusted model (IRR 0.594, p = 0.728) and the unadjusted model (IRR 0.637, p = 0.752) on the same rows are well behaved. Trio status is near-constant in the populations those worksheets use, the unfiltered cohorts, where 109 of 113 patients were sequenced as trios and 99 of 101 in HC80, leaving four and two non-trio patients; the 90-of-93 figure belongs to the global informative-WES exclusion cohort, which those worksheets do not use. A full model that is estimable and regular keeps its usual interpretation; it is the boundary cases, where the trio indicator is nearly collinear with the group indicator, that reduce to the contrast within the trio-sequenced patients. This is recorded in the method freeze, under Table S16 (continued) and in worksheet 61, where 167 of 1,587 primary comparisons have an estimable sex/age model but no estimable full model, and 50 have neither.

One rule, applied everywhere. A model with no estimate carries no p and no q value and does not enter the Benjamini–Hochberg family of its cohort and endpoint. This was verified to hold, cell by cell, in worksheets 24, 30, 41, 42 and 61, and the step-up itself was recomputed from the p values for all four endpoints in every family.

Because the rule removes different comparisons from different endpoints, the denominator of the correction differs between endpoints within the same family. The denominators are given explicitly in worksheets 25 and 31 and under Table S9, whose “Comparisons” column counts the group–pathway comparisons of the family rather than the denominator of the correction.

Primary pathway-specific family	Comparisons	MW	Poisson	Fisher	Logistic
Profile, ALL	224	224	218	224	141
Profile, HC80	190	190	184	190	122
Diagnosis, ALL	603	603	589	603	409
Diagnosis, HC80	570	570	556	570	382

The FDR-positive counts are identical whether the family size is taken as the number of estimable tests or as the total number of comparisons, so the rule changes no reported conclusion.

7. Gene-level contribution against sequence opportunity

For the principal profile signal the gene-level decomposition was rebuilt from the variant exports and matched to the external reference annotations, giving 42 observed member genes with a MANE Select CDS length and a Samocha missense mutability estimate (worksheet 60).

Gene-level quantity	CDS length (MANE)	Missense mutability
Total VUS count per gene	ρ = 0.50, p = 0.0007	ρ = 0.50, p = 0.0007
C3 excess rate per gene	ρ = −0.06, p = 0.72	ρ = −0.04, p = 0.80

Total counts behave as a length- and opportunity-driven quantity, as expected. No correlation was detected between the case-minus-control excess and either annotation; across 42 genes that absence does not exclude confounding by mutation opportunity, and the excess is a difference of mean per-patient counts rather than a quantity normalized by each patient total VUS as the Poisson offset is. These are gene-level correlations and not a burden model adjusted for

individual sequencing opportunity, which the frozen data do not support; the manuscript states this limitation and Table S22 carries the same note.

8. Cross-framework comparison of the principal pathway

Transcriptional Regulation by MECP2 is the one pathway nominal in both frameworks, and the two groupings in which it emerges — profile C3 and diagnosis ADHD — are not paired with each other in the design. The two were therefore refitted head to head on identical patients, with the same offset model, on the pathway-specific primary cohorts and on two sensitivity cohorts. The marginal rows of the primary cohorts reproduce worksheets 24 and 30 exactly, and those of the unfiltered cohorts reproduce the circularity-audit rows of the same worksheets, so the comparison rests on the published estimates. The outlier-trimmed cohorts of worksheet 62 are named "primary, no outliers" because they keep the pathway-specific circularity exclusion before trimming (n = 104 and 92); they are not the ALL_NO_OUTLIERS and HC80_NO_OUTLIERS scopes of worksheets 24 and 30 (n = 106 and 94), which trim the unfiltered cohort, and their estimates are not expected to match those rows.

Cohort	C3 alone	ADHD alone	C3 adjusted for ADHD	ADHD adjusted for C3
ALL, n = 111	1.462 (p = 0.022)	1.424 (p = 0.034)	1.424 (p = 0.030)	1.384 (p = 0.044)
HC80, n = 99	1.530 (p = 0.019)	1.423 (p = 0.059)	1.482 (p = 0.027)	1.367 (p = 0.083)

The two labels identify largely different patients — 16 of 63 labeled patients carry both in ALL, Jaccard 0.25, Fisher p = 0.42 — although a non-significant overlap test does not establish independence. Each estimate remains nominal after adjustment for the other in ALL, whereas in HC80 only C3 does (ADHD p = 0.083). The frameworks were not shown to differ, and the verification is explicit on this point: the Wald test of the difference between the two coefficients in the joint model gives a ratio of 1.03 (95% CI 0.66–1.59; p = 0.90) in ALL and 1.08 (0.67–1.77; p = 0.75) in HC80, so the fact that one grouping reaches $p<0.05$ in a cohort where the other does not is a difference in significance and not a demonstrated difference in effect. The four-way cross-classification places the highest estimate on the patients carrying both labels (IRR 2.00, 95% CI 1.26–3.17), but the C3 × ADHD interaction is not significant either (p = 0.74 in ALL, 0.70 in HC80), so that estimate is compatible with a multiplicative combination of the two labels. ASD, the diagnosis matched to C3, runs the other way and does so more clearly once C3 is accounted for. Both estimates attenuate under sex/age adjustment (C3 p = 0.053, ADHD p = 0.063 in ALL) and under full adjustment, and in the outlier-trimmed cohorts; dispersion ranges from 0.85 to 1.04 over all models and from 0.86 to 0.98 over the joint models. The pathway was selected because it was already nominal in both frameworks, so the comparison is conditioned on that selection: it carries no q value, enters no correction family and supports no confirmatory claim. Worksheet 62 carries every model and cohort, summarized in Supplementary Table S23, together with the seed and the resampling and permutation schemes needed to reproduce the bootstrap and permutation results exactly.

9. Internal consistency of the statistical report

The following relations were recomputed from the worksheets themselves across all 7,935 comparison rows of worksheets 24 and 30, in every cohort and scope.

Check	Outcome
Carrier counts against group sizes; carrier percentages and their differences	No discrepancy
Mean pathway-VUS counts against event totals and group sizes	No discrepancy
Eligible sample sizes against the declared ranges (107–113; 96–101)	No discrepancy
Benjamini–Hochberg step-up recomputed from the p values, all four endpoints	Reproduced exactly
Per-endpoint correction denominators in worksheets 25 and 31	Reproduced exactly
Agreement between each Poisson interval and its p value at the 5% level	No discrepancy
Direction labels against the published incidence-rate ratios	No discrepancy
Derived significance flags and result labels	No discrepancy
Duplicate cohort/group/pathway comparisons	None present

Check	Outcome
Hypergeometric convergence p values and both correction families, worksheets 22, 23, 28, 29	Reproduced exactly
Covariate-family summary in worksheet 43 against worksheets 41 and 42	Reproduced exactly
Unadjusted columns of worksheet 61 against worksheets 24 and 30	Agree to better than 1×10^{-8}
Marginal rows of worksheet 62 against worksheets 24 and 30	Identical to six decimals
Divergent or implausible estimates in the primary Poisson burden models	None present

10. Consistency across documents

Object	Outcome
116 numeric claims in the manuscript: model fit, class sizes, diagnostic prevalences, anchor genes, convergence counts, every incidence-rate ratio, interval, p and q cited, covariate checks and the BCH sensitivity	All agree with the workbook
Tables S10, S11 and S12 against worksheets 24 and 30, cell by cell: effect estimates, intervals, p, q, Fisher p, adjusted logistic p, gene counts, hierarchy level, group sizes, carriers and mean counts	No discrepancy beyond the two-decimal rounding of the printed tables
Table S8 against worksheets 22 and 23, both correction families	No discrepancy
Table S23 and the cross-framework paragraph of the manuscript against worksheet 62	No discrepancy
Table S16 against worksheet 61; Table S16 (continued) against worksheet 43	No discrepancy
Table S17 against worksheet 47	164 of 164 rows agree
Tables S2, S3, S4, S5, S6, S7, S9, S13, S18, S20, S21, S22 against their worksheets	No discrepancy
Patient-level report and source clinical database against the statistical report: 128 identifiers, latent class assignments, posterior probabilities, cohort flags, BCH weights	No discrepancy
Quality-control reconciliations declared in worksheet 04 and Table S20	All pass

11. Cohort definitions used by each worksheet

Three cohort definitions coexist in the workbook and are distinguished explicitly in each worksheet header. Worksheets 24, 25, 30 and 31 carry the pathway-specific primary analysis and its named sensitivity cohorts, and worksheets 59 to 62 are computed on those same primary cohorts, worksheet 62 also on outlier-trimmed versions of those same primary cohorts (n = 104 and 92, not to be confused with the 106 and 94 of the no-outlier scopes) and on the unfiltered cohorts. Worksheets 41, 42 and 48 to 52 are computed on the unfiltered cohort (n = 113 and 101); for the C3–MECP2 comparison their group sizes are 38/75 and 36/65, against 37/74 and 35/64 for the primary cohorts. Supplementary Table S19 is computed on the global informative-WES exclusion cohort (n = 93 and 84), for which the same comparison has group sizes 29/64 and 27/57. Each of these correspondences was checked against the underlying group sizes and effect estimates.

12. Limits of the verification

Four elements of the analysis were taken as given rather than reproduced from source. The latent class model itself was not refitted; the class assignments and posterior probabilities were instead checked for consistency across the statistical report, the patient-level report and the clinical database. The Reactome v97 gene-set archive was not re-downloaded, so pathway membership was taken from the frozen catalog; the member genes carrying events in the refitted comparisons were reconstructed from the variant exports. The binary symptom indicators were taken as coded in the clinical database and not re-extracted from clinical records. Finally, the independent refit covers the Poisson burden endpoint on the pathway-specific primary cohorts, its covariate-adjusted versions, the leave-one-

gene-out analysis and the cross-framework comparison; the Mann–Whitney, Fisher and carrier-logistic endpoints, the negative-binomial models and the named sensitivity scopes were verified through the internal and cross-document checks of sections 9 and 10 rather than by refitting.

13. Conclusion

The variant pipeline reproduces exactly, including the number of rows removed at each declared filtering step. Every one of the 1,587 pathway-specific primary comparisons reproduces from the raw exports, with identical group sizes and estimates agreeing to better than one part in a hundred million, and the principal signal reproduces to six decimal places on every cohort examined, as do its covariate-adjusted and leave-one-gene-out variants. The internal relations of the statistical report reconcile without exception, the supplementary tables agree with their source worksheets, and every numeric claim in the manuscript agrees with the workbook. Comparisons that cannot support inference are reported as non-estimable under a single rule, with the diagnosis confirmed case by case, the correction denominators are stated per endpoint, the anchor-convergence statistic is accompanied by both of its correction families, and the cross-framework comparison of the principal pathway is reported with the selection it is conditioned on made explicit; all three are used descriptively only.
