# Supplementary material for: Integrating a Transdiagnostic Clinical Approach with Whole-Exome Sequencing in Neurodevelopmental Disorders: A Single-Center Exploratory Study
Source: Diagnostics (Basel). 2026 Nov 6;16(21):3674. doi: 10.3390/diagnostics16213674 (PMC13763132; doi:10.3390/diagnostics16213674)
Supplement: Supplementary file 1 [file diagnostics-16-03674-s001.zip › Supplementary_materials.pdf]

Supplementary Materials

Integrating a Transdiagnostic Clinical Approach with Whole-Exome Sequencing in Neurodevelopmental Disorders: A Single-Center Exploratory Study

Supplementary Tables

Table S1. Complete list of 26 candidate symptom indicators: domain, HPO/source definition, prevalence, and inclusion in the 23-indicator LCA

Domain	Indicator	HPO/source definition	Yes	Observed	Prevalence	K=4 status	Reason
NDD	Expressive language delay	Expressive language delay HP:0002474 (2: Yes, 1: No)	101	128	78.9%	Retained	
NDD	Delayed walking	Delayed ability to walk HP:0031936 (2: Yes, 1: No)	41	128	32.0%	Retained	
NDD	Developmental regression	Developmental regression HP:0002376 (2: Yes, 1: No)	21	128	16.4%	Retained	
NDD	Intellectual disability	Intellectual disability HP:0001249 (2: Yes, 1: No, 999: Inconsistent)	75	128	58.6%	Retained	
NDD	Short attention span	Short attention span HP:0000736 (2: Yes, 1: No)	102	128	79.7%	Retained	
NDD	Hyperactivity	Hyperactivity HP:0000752 (2: Yes, 1: No)	77	128	60.2%	Retained	
NDD	Impulsivity	Impulsivity HP:0100710 (2: Yes, 1: No)	68	128	53.1%	Retained	
NDD	Sensory processing abnormality	Sensory behavioral abnormality HP:5200046 (2: Yes, 1: No)	77	128	60.2%	Retained	
NDD	Abnormal social interaction	Abnormal social development HP:0025732 (2: Yes, 1: No)	74	128	57.8%	Retained	
NDD	Motor stereotypy	Motor stereotypy HP:0000733 (2: Yes, 1: No)	61	128	47.7%	Retained	
NDD	Restricted/repetitive behaviors	Restricted or repetitive behaviors or interests HP:0031432 (2: Yes, 1: No)	38	128	29.7%	Retained	
NDD	Clumsiness	Clumsiness HP:0002312 (2: Yes, 1: No)	58	128	45.3%	Retained	
NDD	Verbal dyspraxia	Verbal dyspraxia HP:0011098 (2: Yes, 1: No)	16	128	12.5%	Retained	
Psychiatric	Aggressive behavior	Aggressive behavior HP:0000718 (2: Yes, 1: No)	64	128	50.0%	Retained	
Psychiatric	Irritability	Irritability HP:0000737 (2: Yes, 1: No)	39	128	30.5%	Retained	
Psychiatric	Anxiety	Anxiety HP:0000739 (2: Yes, 1: No)	48	128	37.5%	Retained	
Psychiatric	Psychotic episodes	Psychotic episodes HP:0000725 (2: Yes, 1: No)	1	128	0.8%	Excluded	Prevalence <5% in common estimation sample
Psychiatric	Obsessive-compulsive trait	Obsessive-compulsive trait HP:0008770 (2: Yes, 1: No)	8	128	6.2%	Retained	
Psychiatric	Abnormal emotional state	Abnormal emotional state HP:0100851 (2: Yes, 1: No)	8	128	6.2%	Retained	
Neurologic	Involuntary movements	Involuntary movements HP:0004305 (2: Yes, 1: No)	12	128	9.4%	Retained	
Neurologic	Ataxia	Ataxia HP:0001251 (2: Yes, 1: No)	10	128	7.8%	Retained	
Neurologic	Hearing impairment	Hearing impairment HP:0000365 (2: Yes, 1: No)	5	128	3.9%	Excluded	Prevalence <5% in common estimation sample
Neurologic	Pyramidal signs	Abnormal pyramidal sign HP:0007256 (2: Yes, 1: No)	10	128	7.8%	Retained	
Neurologic	Extrapyramidal motor abnormality	Abnormality of extrapyramidal motor function HP:0002071 (2: Yes, 1: No)	2	128	1.6%	Excluded	Prevalence <5% in common estimation sample
Neurologic	Hypotonia	Hypotonia HP:0001252 (2: Yes, 1: No)	9	128	7.0%	Retained	
Neurologic	Seizures	Seizure HP:0001250 (2: Yes, 1: No)	16	128	12.5%	Retained	

Table S2. LCA model-selection sequence (23 indicators)

K	Parameters	LL	AIC	BIC	saBIC	Entropy R^2	Mean max posterior	Hard sizes (optimizer order)	Smallest n
1	23	-1508.0954	3062.1908	3127.7875	3055.0489	1.000	1.000	128	128
2	47	-1442.8720	2979.7439	3113.7894	2965.1495	0.865	0.963	84, 44	44
3	71	-1405.3149	2952.6299	3155.1240	2930.5830	0.863	0.942	37, 21, 70	21
4	95	-1375.0287	2940.0573	3211.0002	2910.5580	0.866	0.938	34, 30, 23, 41	23
5	119	-1345.8219	2929.6439	3269.0355	2892.6921	0.897	0.943	35, 36, 21, 31, 5	5
6	143	-1318.6805	2923.3609	3331.2013	2878.9567	0.927	0.953	27, 5, 17, 36, 15, 28	5

Note: component order for candidate models is optimizer-specific. K=4 labels were clinically aligned after fitting, producing C1 n=34, C2 n=30, C3 n=41, and C4 n=23. Candidate models are shown for model selection.

Table S3. K=4 diagnostics and bootstrap stability

Diagnostic	Result
Observed log-likelihood	-1375.0287
Observed-information rank	95/95
Information condition number	1.769e+07
Boundary probabilities	12
Largest bivariate residual	9.384
Largest-residual pair	Expressive language delay + Intellectual disability
Pairwise p	0.025695456561688108
Max-residual FWER p	0.892
BLRT 3→4	LRT 60.573; p=0.020
BLRT 4→5	LRT 58.413; p=0.020
Bootstrap ARI	median 0.315; IQR 0.239–0.394
Bootstrap fits with smallest class <10	42.4%

Table S4. K=4 conditional probabilities

Domain	Indicator	Overall	C1	C2	C3	C4
NDD	Expressive language delay	78.9%	0.901	0.641	0.840	0.727
NDD	Delayed walking	32.0%	0.286	0.115	0.279	0.693
NDD	Developmental regression	16.4%	0.217	0.000	0.326	0.000
NDD	Intellectual disability	58.6%	0.667	0.452	0.591	0.631
NDD	Short attention span	79.7%	0.503	1.000	0.880	0.795
NDD	Hyperactivity	60.2%	0.043	0.831	0.904	0.526
NDD	Impulsivity	53.1%	0.000	0.874	0.705	0.517
NDD	Sensory processing abnormality	60.2%	0.597	0.689	0.760	0.212
NDD	Abnormal social interaction	57.8%	0.610	0.785	0.680	0.097
NDD	Motor stereotypy	47.7%	0.370	0.428	0.770	0.147
NDD	Restricted/repetitive behaviors	29.7%	0.151	0.242	0.578	0.049
NDD	Clumsiness	45.3%	0.473	0.411	0.311	0.737
NDD	Verbal dyspraxia	12.5%	0.165	0.000	0.131	0.212
Psychiatric	Aggressive behavior	50.0%	0.368	0.831	0.505	0.266
Psychiatric	Irritability	30.5%	0.159	0.804	0.228	0.033
Psychiatric	Anxiety	37.5%	0.318	0.889	0.127	0.277
Psychiatric	Obsessive-compulsive trait	6.2%	0.000	0.141	0.062	0.052
Psychiatric	Abnormal emotional state	6.2%	0.092	0.035	0.048	0.082
Neurologic	Involuntary movements	9.4%	0.031	0.156	0.029	0.220
Neurologic	Ataxia	7.8%	0.000	0.000	0.068	0.298
Neurologic	Pyramidal signs	7.8%	0.000	0.069	0.026	0.289
Neurologic	Hypotonia	7.0%	0.000	0.000	0.028	0.329

Domain	Indicator	Overall	C1	C2	C3	C4
Neurologic	Seizures	12.5%	0.000	0.137	0.160	0.215

Table S5. K=4 class sizes and assignment quality

Class	Profile	Assigned n	Assigned %	Mean posterior	Median posterior	HC80 n	HC80 % within class
1	Cognitive–Language	34	26.6%	0.921	0.967	29	85.3%
2	Executive–Psychiatric	30	23.4%	0.925	0.979	25	83.3%
3	Social–Sensory/Repetitive	41	32.0%	0.959	0.990	39	95.1%
4	Motor–Neurological	23	18.0%	0.943	0.996	21	91.3%

Table S6. Patients with a VUS file and pathway-specific eligible sample-size ranges

Class	Profile	Clinical n	With a VUS file, n	Primary eligible N range	HC80 with a VUS file, n	HC80 eligible N range
1	Cognitive–Language	34	29	109–113	25	97–101
2	Executive–Psychiatric	30	26	107–113	21	98–101
3	Social–Sensory/Repetitive	41	38	107–113	36	96–101
4	Motor–Neurological	23	20	107–112	19	96–100

Table S7. Profile anchor genes, patients with a VUS file, and pathway counts

Class	Profile	With a VUS file, n	ALL anchors	Mapped	Pathways	Convergent	HC80 anchors
1	Cognitive–Language	29	*ANKRD11*; *KAT6A*; *PRKAR1B*; *SETBP1*; *TRIP12*	3	61	0	*ANKRD11*; *PRKAR1B*; *TRIP12*
2	Executive–Psychiatric	26	*ADNP*; *ATRX*; *KMT2C*	3	41	1	*ADNP*; *ATRX*
3	Social–Sensory/Repetitive	38	*CLCN4*; *L1CAM*; *LZTR1*; *MBD5*; *MED13L*; *MYT1L*; *NLGN1*; *SCN2A*; *SHANK3*; *SMPD4*; *TBL1XR1*; *TRRAP*	10	97	23	*CLCN4*; *L1CAM*; *LZTR1*; *MBD5*; *MED13L*; *MYT1L*; *NLGN1*; *SCN2A*; *SHANK3*; *SMPD4*; *TBL1XR1*; *TRRAP*
4	Motor–Neurological	20	*AUTS2*; *PHIP*; *PURA*; *RBFOX1*; *SRCAP*; *TCF4*; *TMEM240*	4	25	4	*AUTS2*; *PHIP*; *PURA*; *RBFOX1*; *TCF4*; *TMEM240*

Table S8. All convergent profile anchor pathways

Cohort	Class	Profile	Reactome ID	Pathway	Genes	Hierarchy level	Anchor genes	n anchors	>1,000	Levels 0–1	p	q (convergence family)	q (anchor-pathway family)
ALL	2	Executive–Psychiatric	R-HSA-4839726	Chromatin organization	346	0	*ADNP*; *KMT2C*	2	0	1	0.0024	0.0121	0.0498
ALL	3	Social–Sensory/Repetitive	R-HSA-422475	Axon guidance	542	2	*L1CAM*; *SCN2A*; *SHANK3*	3	0	0	0.0087	0.0221	0.0571
ALL	3	Social–Sensory/Repetitive	R-HSA-9675108	Nervous system development	568	1	*L1CAM*; *SCN2A*; *SHANK3*	3	0	1	0.0099	0.0231	0.0571
ALL	3	Social–Sensory/Repetitive	R-HSA-556833	Metabolism of lipids	759	1	*MED13L*; *SMPD4*; *TBL1XR1*	3	0	1	0.0217	0.0405	0.0702
ALL	3	Social–Sensory/Repetitive	R-HSA-1266738	Developmental Biology	1473	0	*L1CAM*; *MED13L*; *SCN2A*; *SHANK3*; *TBL1XR1*	5	1	1	0.0041	0.0121	0.0530
ALL	3	Social–Sensory/Repetitive	R-HSA-445095	Interaction between L1 and Ankyrins	31	4	*L1CAM*; *SCN2A*	2	0	0	0.0003	0.0081	0.0187
ALL	3	Social–Sensory/Repetitive	R-HSA-1989781	*PPARA* activates gene expression	117	3	*MED13L*; *TBL1XR1*	2	0	0	0.0040	0.0121	0.0530
ALL	3	Social–Sensory/Repetitive	R-HSA-373760	*L1CAM* interactions	121	3	*L1CAM*; *SCN2A*	2	0	0	0.0043	0.0121	0.0530
ALL	3	Social–Sensory/Repetitive	R-HSA-6794361	Neurexins and neuroligins	57	2	*NLGN1*; *SHANK3*	2	0	0	0.0010	0.0121	0.0312
ALL	3	Social–Sensory/Repetitive	R-HSA-381340	Transcriptional regulation of white adipocyte differentiation	84	2	*MED13L*; *TBL1XR1*	2	0	0	0.0021	0.0121	0.0498
ALL	3	Social–Sensory/Repetitive	R-HSA-400206	Regulation of lipid metabolism by PPARalpha	119	2	*MED13L*; *TBL1XR1*	2	0	0	0.0042	0.0121	0.0530
ALL	3	Social–Sensory/Repetitive	R-HSA-5688426	Deubiquitination	269	2	*MBD5*; *TRRAP*	2	0	0	0.0201	0.0401	0.0702
ALL	3	Social–Sensory/Repetitive	R-HSA-9824446	Viral Infection Pathways	1269	2	*MED13L*; *TBL1XR1*	2	1	0	0.2868	0.3491	0.3261
ALL	3	Social–Sensory/Repetitive	R-HSA-6794362	Protein-protein interactions at synapses	88	1	*NLGN1*; *SHANK3*	2	0	1	0.0023	0.0121	0.0498
ALL	3	Social–Sensory/Repetitive	R-HSA-9843745	Adipogenesis	110	1	*MED13L*; *TBL1XR1*	2	0	1	0.0036	0.0121	0.0530
ALL	3	Social–Sensory/Repetitive	R-HSA-597592	Post-translational protein modification	1475	1	*MBD5*; *TRRAP*	2	1	1	0.3537	0.4126	0.3884
ALL	3	Social–Sensory/Repetitive	R-HSA-392499	Metabolism of proteins	2133	0	*MBD5*; *TRRAP*	2	1	1	0.5542	0.5969	0.5669
ALL	3	Social–Sensory/Repetitive	R-HSA-1430728	Metabolism	2197	0	*MED13L*; *SMPD4*; *TBL1XR1*	3	1	1	0.2725	0.3468	0.3146
ALL	3	Social–Sensory/Repetitive	R-HSA-5663205	Infectious disease	1696	1	*MED13L*; *TBL1XR1*	2	1	1	0.4242	0.4751	0.4568
ALL	3	Social–Sensory/Repetitive	R-HSA-112316	Neuronal System	412	0	*NLGN1*; *SHANK3*	2	0	1	0.0442	0.0728	0.0884
ALL	3	Social–Sensory/Repetitive	R-HSA-3247509	Chromatin modifying enzymes	238	1	*TBL1XR1*; *TRRAP*	2	0	1	0.0159	0.0343	0.0673
ALL	3	Social–Sensory/Repetitive	R-HSA-1643685	Disease	2512	0	*MED13L*; *TBL1XR1*	2	1	1	0.6526	0.6768	0.6645
ALL	3	Social–Sensory/Repetitive	R-HSA-162582	Signal Transduction	2622	0	*TBL1XR1*; *TRRAP*	2	1	1	0.6784	0.6784	0.6845
ALL	3	Social–Sensory/Repetitive	R-HSA-4839726	Chromatin organization	346	0	*TBL1XR1*; *TRRAP*	2	0	1	0.0321	0.0562	0.0808
ALL	4	Motor–Neurological	R-HSA-212436	Generic Transcription Pathway	1230	2	*AUTS2*; *RBFOX1*	2	1	0	0.0549	0.0853	0.0953
ALL	4	Motor–Neurological	R-HSA-73857	RNA Polymerase II Transcription	1322	1	*AUTS2*; *RBFOX1*	2	1	1	0.0627	0.0924	0.1025
ALL	4	Motor–Neurological	R-HSA-74160	Gene expression (Transcription)	1550	0	*AUTS2*; *RBFOX1*	2	1	1	0.0839	0.1174	0.1240
ALL	4	Motor–Neurological	R-HSA-162582	Signal Transduction	2622	0	*PHIP*; *TCF4*	2	1	1	0.2103	0.2804	0.2513
HC80	3	Social–Sensory/Repetitive	R-HSA-1266738	Developmental Biology	1473	0	*L1CAM*; *MED13L*; *SCN2A*; *SHANK3*; *TBL1XR1*	5	1	1	0.0041	0.0129	0.0410
HC80	3	Social–Sensory/Repetitive	R-HSA-422475	Axon guidance	542	2	*L1CAM*; *SCN2A*; *SHANK3*	3	0	0	0.0087	0.0235	0.0447
HC80	3	Social–Sensory/Repetitive	R-HSA-9675108	Nervous system development	568	1	*L1CAM*; *SCN2A*; *SHANK3*	3	0	1	0.0099	0.0243	0.0459
HC80	3	Social–Sensory/Repetitive	R-HSA-556833	Metabolism of lipids	759	1	*MED13L*; *SMPD4*; *TBL1XR1*	3	0	1	0.0217	0.0419	0.0589
HC80	3	Social–Sensory/Repetitive	R-HSA-445095	Interaction between L1 and Ankyrins	31	4	*L1CAM*; *SCN2A*	2	0	0	0.0003	0.0078	0.0106
HC80	3	Social–Sensory/Repetitive	R-HSA-1989781	*PPARA* activates gene expression	117	3	*MED13L*; *TBL1XR1*	2	0	0	0.0040	0.0129	0.0410
HC80	3	Social–Sensory/Repetitive	R-HSA-373760	*L1CAM* interactions	121	3	*L1CAM*; *SCN2A*	2	0	0	0.0043	0.0129	0.0410
HC80	3	Social–Sensory/Repetitive	R-HSA-6794361	Neurexins and neuroligins	57	2	*NLGN1*; *SHANK3*	2	0	0	0.0010	0.0129	0.0265

Cohort	Class	Profile	Reactome ID	Pathway	Genes	Hierarchy level	Anchor genes	n anchors	>1,000	Levels 0–1	p	q (convergence family)	q (anchor-pathway family)
HC80	3	Social–Sensory/Repetitive	R-HSA-381340	Transcriptional regulation of white adipocyte differentiation	84	2	*MED13L*; *TBL1XR1*	2	0	0	0.0021	0.0129	0.0399
HC80	3	Social–Sensory/Repetitive	R-HSA-400206	Regulation of lipid metabolism by PPARalpha	119	2	*MED13L*; *TBL1XR1*	2	0	0	0.0042	0.0129	0.0410
HC80	3	Social–Sensory/Repetitive	R-HSA-5688426	Deubiquitination	269	2	*MBD5*; *TRRAP*	2	0	0	0.0201	0.0417	0.0583
HC80	3	Social–Sensory/Repetitive	R-HSA-9824446	Viral Infection Pathways	1269	2	*MED13L*; *TBL1XR1*	2	1	0	0.2868	0.3520	0.3205
HC80	3	Social–Sensory/Repetitive	R-HSA-6794362	Protein-protein interactions at synapses	88	1	*NLGN1*; *SHANK3*	2	0	1	0.0023	0.0129	0.0399
HC80	3	Social–Sensory/Repetitive	R-HSA-9843745	Adipogenesis	110	1	*MED13L*; *TBL1XR1*	2	0	1	0.0036	0.0129	0.0410
HC80	3	Social–Sensory/Repetitive	R-HSA-3247509	Chromatin modifying enzymes	238	1	*TBL1XR1*; *TRRAP*	2	0	1	0.0159	0.0358	0.0540
HC80	3	Social–Sensory/Repetitive	R-HSA-597592	Post-translational protein modification	1475	1	*MBD5*; *TRRAP*	2	1	1	0.3537	0.4152	0.3884
HC80	3	Social–Sensory/Repetitive	R-HSA-392499	Metabolism of proteins	2133	0	*MBD5*; *TRRAP*	2	1	1	0.5542	0.5986	0.5692
HC80	3	Social–Sensory/Repetitive	R-HSA-1430728	Metabolism	2197	0	*MED13L*; *SMPD4*; *TBL1XR1*	3	1	1	0.2725	0.3503	0.3063
HC80	3	Social–Sensory/Repetitive	R-HSA-5663205	Infectious disease	1696	1	*MED13L*; *TBL1XR1*	2	1	1	0.4242	0.4772	0.4478
HC80	3	Social–Sensory/Repetitive	R-HSA-112316	Neuronal System	412	0	*NLGN1*; *SHANK3*	2	0	1	0.0442	0.0746	0.0841
HC80	3	Social–Sensory/Repetitive	R-HSA-4839726	Chromatin organization	346	0	*TBL1XR1*; *TRRAP*	2	0	1	0.0321	0.0578	0.0726
HC80	3	Social–Sensory/Repetitive	R-HSA-1643685	Disease	2512	0	*MED13L*; *TBL1XR1*	2	1	1	0.6526	0.6777	0.6667
HC80	3	Social–Sensory/Repetitive	R-HSA-162582	Signal Transduction	2622	0	*TBL1XR1*; *TRRAP*	2	1	1	0.6784	0.6784	0.6856
HC80	4	Motor–Neurological	R-HSA-212436	Generic Transcription Pathway	1230	2	*AUTS2*; *RBFOX1*	2	1	0	0.0549	0.0871	0.0891
HC80	4	Motor–Neurological	R-HSA-73857	RNA Polymerase II Transcription	1322	1	*AUTS2*; *RBFOX1*	2	1	1	0.0627	0.0940	0.0968
HC80	4	Motor–Neurological	R-HSA-74160	Gene expression (Transcription)	1550	0	*AUTS2*; *RBFOX1*	2	1	1	0.0839	0.1192	0.1207
HC80	4	Motor–Neurological	R-HSA-162582	Signal Transduction	2622	0	*PHIP*; *TCF4*	2	1	1	0.2103	0.2839	0.2444

Note: a pathway enters this table only when it already contains at least two anchor genes, and the hypergeometric statistic is driven by that same count, so the correction is applied within a set defined by the statistic it corrects. Both families are given: the convergence family corrects over the 28 selected rows (27 in HC80), the anchor-pathway family over every anchor pathway of the cohort (224 rows, 190 in HC80). In the ALL cohort 15 rows reach q<0.05 under the first and 5 under the second. Anchor convergence is reported descriptively and neither column is used for inference; statistical-report worksheets 22 and 23 carry both.

Table S9. Multiplicity families for pathway-specific primary/HC80 analyses, global informative-WES exclusion sensitivities, unfiltered-cohort circularity audits, no-outlier sensitivities, and strict WES-negative sensitivities.

Framework	Cohort	Scope	Comparisons	Poisson p<.05	Poisson q<.05	Fisher p<.05	Fisher q<.05	Logistic p<.05	Logistic q<.05	MW p<.05	MW q<.05
Profile	ALL	pathway-specific primary	224	4	0	4	0	5	0	10	0
Profile	HC80	pathway-specific primary	190	7	0	2	0	2	0	4	0
Diagnosis	ALL	pathway-specific primary	603	32	0	20	0	20	0	37	0
Diagnosis	HC80	pathway-specific primary	570	22	0	17	0	16	0	37	0
Profile	ALL global exclusion	global informative-WES exclusion	224	3	0	0	0	1	0	5	0
Profile	HC80 global exclusion	global informative-WES exclusion	190	4	0	1	0	1	0	5	0
Diagnosis	ALL global exclusion	global informative-WES exclusion	603	37	3	21	0	22	0	48	2
Diagnosis	HC80 global exclusion	global informative-WES exclusion	570	27	1	18	0	11	0	35	3
Profile	ALL unfiltered audit	unfiltered circularity audit	224	5	0	7	0	5	0	11	0
Profile	HC80 unfiltered audit	unfiltered circularity audit	190	8	0	3	0	2	0	6	0
Diagnosis	ALL unfiltered audit	unfiltered circularity audit	603	30	0	22	0	20	0	39	0
Diagnosis	HC80 unfiltered audit	unfiltered circularity audit	570	23	0	16	0	15	0	36	0
Profile	ALL no-outliers	outlier sensitivity	224	4	0	2	0	4	0	5	0
Profile	HC80 no-outliers	outlier sensitivity	190	11	1	2	0	2	0	6	0
Diagnosis	ALL no-outliers	outlier sensitivity	603	28	0	22	0	19	0	37	0
Diagnosis	HC80 no-outliers	outlier sensitivity	570	30	0	15	0	14	0	36	0
Profile	ALL WES0	strict WES-negative sensitivity	224	4	0	3	0	4	0	4	0

Framework	Cohort	Scope	Comparisons	Poisson p<.05	Poisson q<.05	Fisher p<.05	Fisher q<.05	Logistic p<.05	Logistic q<.05	MW p<.05	MW q<.05
Profile	HC80 WES0	strict WES-negative sensitivity	190	7	0	2	0	3	0	4	0
Diagnosis	ALL WES0	strict WES-negative sensitivity	603	35	4	22	0	18	0	46	2
Diagnosis	HC80 WES0	strict WES-negative sensitivity	570	28	0	23	0	16	0	35	0

Note: the Comparisons column counts the group–pathway comparisons of the family, not the denominator of the Benjamini–Hochberg correction, which is the number of estimable tests of each endpoint and therefore differs between endpoints. For the pathway-specific primary families those denominators are, for Mann–Whitney, Poisson, Fisher and logistic: profile ALL 224, 218, 224, 141; profile HC80 190, 184, 190, 122; diagnosis ALL 603, 589, 603, 409; diagnosis HC80 570, 556, 570, 382. They are given for every family in statistical-report worksheets 25 and 31. Logistic counts refer to carrier-status logistic regression adjusted for standardized total VUS. A comparison showing complete or quasi-complete separation — a zero cell in the 2×2 carrier table or a divergent coefficient — does not support valid inference: it is reported as unavailable and does not enter the family. Thirty profile and 98 diagnosis carrier models fall in this category. No logistic comparison is FDR-significant in any cohort. Poisson, Mann–Whitney, Fisher and BCH results are unaffected. A comparison in which every eligible patient is a carrier is handled differently by the two carrier endpoints: the Fisher test returns p=1 and the comparison stays in the Fisher family, whereas the adjusted logistic model has no estimate, carries no p or q value and is excluded from the logistic family. This is why the logistic denominators above are the smallest of the four.

Table S10. All nominal profile Poisson signals, both directions (pathway-specific primary and HC80)

Cohort	Class	Profile	ID	Pathway	Genes	Hierarchy level	Direction	n case/control	Mean count	IRR (95% CI)	p	q	Carriers	Fisher p	Adjusted logistic p
ALL	1	Cognitive–Language	R-HSA-5358351	Signaling by Hedgehog	137	1	Lower	29/84 (N=113)	1.59/2.49	0.68 (0.51–0.91)	0.0092	0.9214	26/29 vs 74/84	1.0000	0.7716
ALL	3	Social–Sensory/Repetitive	R-HSA-9717189	Sensory perception of taste	47	1	Lower	38/75 (N=113)	1.11/1.79	0.62 (0.42–0.92)	0.0164	0.9214	27/38 vs 57/75	0.6500	0.5486
ALL	3	Social–Sensory/Repetitive	R-HSA-8986944	Transcriptional Regulation by *MECP2*	63	3	Higher	37/74 (N=111)	1.65/1.12	1.46 (1.06–2.03)	0.0223	0.9214	29/37 vs 50/74	0.2728	0.2692
ALL	3	Social–Sensory/Repetitive	R-HSA-9717207	Sensory perception of sweet, bitter, and umami (glutamate) taste	41	2	Lower	38/75 (N=113)	1.00/1.53	0.65 (0.43–0.99)	0.0471	0.9214	25/38 vs 53/75	0.6684	0.5485
HC80	1	Cognitive–Language	R-HSA-5358351	Signaling by Hedgehog	137	1	Lower	25/76 (N=101)	1.40/2.46	0.61 (0.45–0.82)	0.0010	0.1882	22/25 vs 67/76	1.0000	0.9510
HC80	1	Cognitive–Language	R-HSA-162582	Signal Transduction	2622	0	Higher	25/72 (N=97)	57.64/57.76	1.08 (1.02–1.14)	0.0135	0.9440	25/25 vs 72/72	1.0000	—
HC80	3	Social–Sensory/Repetitive	R-HSA-8986944	Transcriptional Regulation by *MECP2*	63	3	Higher	35/64 (N=99)	1.66/1.08	1.53 (1.07–2.18)	0.0188	0.9440	27/35 vs 41/64	0.2571	0.2090
HC80	1	Cognitive–Language	R-HSA-372790	Signaling by GPCR	708	1	Higher	25/76 (N=101)	15.80/15.00	1.13 (1.01–1.25)	0.0254	0.9440	25/25 vs 76/76	1.0000	—
HC80	3	Social–Sensory/Repetitive	R-HSA-9717189	Sensory perception of taste	47	1	Lower	36/65 (N=101)	1.17/1.78	0.65 (0.44–0.98)	0.0385	0.9440	27/36 vs 48/65	1.0000	0.9330
HC80	4	Motor–Neurological	R-HSA-525793	Myogenesis	29	1	Higher	18/82 (N=100)	0.89/0.49	1.76 (1.03–3.01)	0.0403	0.9440	11/18 vs 33/82	0.1226	0.1364
HC80	1	Cognitive–Language	R-HSA-5610787	Hedgehog 'off' state	100	2	Lower	25/76 (N=101)	0.88/1.49	0.63 (0.40–1.00)	0.0498	0.9440	15/25 vs 60/76	0.0701	0.0910

Table S11. All positive-direction nominal diagnosis Poisson signals (pathway-specific primary and HC80)

Cohort	Diagnosis	ID	Pathway	Genes	Hierarchy level	>1,000	Levels 0–1	Convergent	n case/control	Mean count	IRR (95% CI)	p	q	Carriers	Fisher p
ALL	ADHD	R-HSA-1430728	Metabolism	2197	0	1	1	1	41/69 (N=110)	50.22/48.86	1.12 (1.05–1.19)	0.0009	0.3528	41/41 vs 69/69	1.0000
ALL	ADHD	R-HSA-9824446	Viral Infection Pathways	1269	2	1	0	1	41/70 (N=111)	17.24/16.21	1.15 (1.04–1.28)	0.0056	0.5207	41/41 vs 70/70	1.0000
ALL	ADHD	R-HSA-556833	Metabolism of lipids	759	1	0	1	1	41/69 (N=110)	25.24/23.23	1.18 (1.05–1.33)	0.0062	0.5207	41/41 vs 69/69	1.0000
ALL	ADHD	R-HSA-9675151	Disorders of Developmental Biology	18	1	0	1	0	42/70 (N=112)	0.60/0.33	1.96 (1.10–3.50)	0.0221	0.8122	19/42 vs 20/70	0.1009
ALL	ADHD	R-HSA-9943965	CHD3, CHD4, CHD5 subfamily	69	3	0	0	0	43/70 (N=113)	0.93/0.64	1.57 (1.05–2.36)	0.0290	0.8122	28/43 vs 33/70	0.0807
ALL	ADHD	R-HSA-8986944	Transcriptional Regulation by *MECP2*	63	3	0	0	0	42/69 (N=111)	1.52/1.16	1.42 (1.03–1.97)	0.0340	0.8280	32/42 vs 47/69	0.3958
ALL	ID	R-HSA-9658195	Leishmania infection	245	3	0	0	0	50/63 (N=113)	5.08/3.97	1.34 (1.07–1.69)	0.0123	0.6220	50/50 vs 59/63	0.1283
ALL	ID	R-HSA-9824443	Parasitic Infection Pathways	245	2	0	0	0	50/63 (N=113)	5.08/3.97	1.34 (1.07–1.69)	0.0123	0.6220	50/50 vs 59/63	0.1283
ALL	ID	R-HSA-432040	Vasopressin regulates renal water homeostasis via Aquaporins	43	2	0	0	0	50/63 (N=113)	0.70/0.37	2.01 (1.16–3.48)	0.0127	0.6220	24/50 vs 20/63	0.0850
ALL	ID	R-HSA-1852241	Organelle biogenesis and maintenance	329	0	0	1	0	49/63 (N=112)	7.39/6.52	1.18 (1.02–1.37)	0.0258	0.8122	49/49 vs 63/63	1.0000

Cohort	Diagnosis	ID	Pathway	Genes	Hierarchy level	>1,000	Levels 0–1	Convergent	n case/control	Mean count	IRR (95% CI)	p	q	Carriers	Fisher p
ALL	ID	R-HSA-164378	PKA activation in glucagon signalling	17	3	0	0	0	50/63 (N=113)	0.56/0.30	1.95 (1.08–3.52)	0.0274	0.8122	22/50 vs 16/63	0.0461
ALL	ID	R-HSA-9660821	ADORA2B mediated anti-inflammatory cytokines production	43	6	0	0	0	50/63 (N=113)	0.66/0.38	1.82 (1.05–3.13)	0.0318	0.8280	24/50 vs 20/63	0.0850
ALL	ID	R-HSA-163615	PKA activation	18	6	0	0	0	50/63 (N=113)	0.56/0.32	1.85 (1.04–3.29)	0.0371	0.8280	22/50 vs 17/63	0.0738
ALL	ID	R-HSA-111931	PKA-mediated phosphorylation of CREB	20	5	0	0	0	50/63 (N=113)	0.56/0.32	1.85 (1.04–3.29)	0.0371	0.8280	22/50 vs 17/63	0.0738
ALL	ID	R-HSA-9662851	Anti-inflammatory response favouring Leishmania parasite infection	159	5	0	0	0	50/63 (N=113)	3.08/2.32	1.39 (1.02–1.91)	0.0397	0.8280	45/50 vs 48/63	0.0814
ALL	ID	R-HSA-9664433	Leishmania parasite growth and survival	159	4	0	0	0	50/63 (N=113)	3.08/2.32	1.39 (1.02–1.91)	0.0397	0.8280	45/50 vs 48/63	0.0814
ALL	ID	R-HSA-445717	Aquaporin-mediated transport	52	1	0	1	0	50/63 (N=113)	0.76/0.48	1.67 (1.02–2.73)	0.0399	0.8280	26/50 vs 25/63	0.2536
ALL	SLD	R-HSA-382551	Transport of small molecules	721	0	0	1	0	6/106 (N=112)	20.67/18.56	1.16 (1.05–1.28)	0.0036	0.5207	6/6 vs 106/106	1.0000
ALL	Sp-Lan-D	R-HSA-1280218	Adaptive Immune System	1159	1	1	1	0	79/32 (N=111)	22.09/19.44	1.15 (1.03–1.27)	0.0093	0.6065	79/79 vs 32/32	1.0000
ALL	Sp-Lan-D	R-HSA-9855142	Cellular responses to mechanical stimuli	103	1	0	1	0	80/33 (N=113)	2.12/1.55	1.39 (1.07–1.81)	0.0143	0.6460	71/80 vs 28/33	0.5459
ALL	Sp-Lan-D	R-HSA-212436	Generic Transcription Pathway	1230	2	1	0	1	79/30 (N=109)	26.00/23.20	1.14 (1.00–1.29)	0.0463	0.8306	79/79 vs 30/30	1.0000
HC80	ADHD	R-HSA-1430728	Metabolism	2197	0	1	1	1	35/63 (N=98)	49.37/48.22	1.13 (1.05–1.21)	0.0008	0.4470	35/35 vs 63/63	1.0000
HC80	ADHD	R-HSA-556833	Metabolism of lipids	759	1	0	1	1	35/63 (N=98)	24.86/22.79	1.20 (1.06–1.37)	0.0051	0.6847	35/35 vs 63/63	1.0000
HC80	ADHD	R-HSA-9824446	Viral Infection Pathways	1269	2	1	0	1	35/64 (N=99)	17.17/16.41	1.15 (1.04–1.28)	0.0067	0.6847	35/35 vs 64/64	1.0000
HC80	ADHD	R-HSA-9943965	CHD3, CHD4, CHD5 subfamily	69	3	0	0	0	37/64 (N=101)	1.00/0.67	1.64 (1.08–2.49)	0.0199	0.7367	25/37 vs 31/64	0.0959
HC80	ADHD	R-HSA-109582	Hemostasis	720	0	0	1	0	37/63 (N=100)	16.11/15.75	1.13 (1.01–1.26)	0.0303	0.8019	37/37 vs 63/63	1.0000
HC80	ID	R-HSA-9658195	Leishmania infection	245	3	0	0	0	46/55 (N=101)	5.20/3.91	1.40 (1.09–1.79)	0.0074	0.6847	46/46 vs 51/55	0.1235
HC80	ID	R-HSA-9824443	Parasitic Infection Pathways	245	2	0	0	0	46/55 (N=101)	5.20/3.91	1.40 (1.09–1.79)	0.0074	0.6847	46/46 vs 51/55	0.1235
HC80	ID	R-HSA-9662851	Anti-inflammatory response favouring Leishmania parasite infection	159	5	0	0	0	46/55 (N=101)	3.17/2.20	1.52 (1.09–2.13)	0.0147	0.7367	41/46 vs 41/55	0.0762
HC80	ID	R-HSA-9664433	Leishmania parasite growth and survival	159	4	0	0	0	46/55 (N=101)	3.17/2.20	1.52 (1.09–2.13)	0.0147	0.7367	41/46 vs 41/55	0.0762
HC80	ID	R-HSA-432040	Vasopressin regulates renal water homeostasis via Aquaporins	43	2	0	0	0	46/55 (N=101)	0.65/0.35	1.99 (1.07–3.68)	0.0286	0.8019	21/46 vs 16/55	0.1001
HC80	SLD	R-HSA-382551	Transport of small molecules	721	0	0	1	0	6/94 (N=100)	20.67/18.34	1.17 (1.06–1.29)	0.0019	0.5416	6/6 vs 94/94	1.0000
HC80	Sp-Lan-D	R-HSA-1280218	Adaptive Immune System	1159	1	1	1	0	69/31 (N=100)	22.03/19.42	1.14 (1.02–1.27)	0.0196	0.7367	69/69 vs 31/31	1.0000
HC80	Sp-Lan-D	R-HSA-9855142	Cellular responses to mechanical stimuli	103	1	0	1	0	70/31 (N=101)	2.10/1.55	1.36 (1.03–1.80)	0.0288	0.8019	63/70 vs 26/31	0.5056

Table S12. All inverse nominal diagnosis Poisson signals (pathway-specific primary and HC80)

Cohort	Diagnosis	ID	Pathway	Genes	Hierarchy level	n case/control	Mean count	IRR (95% CI)	p	q	Anchor genes
ALL	ADHD	R-HSA-9675108	Nervous system development	568	1	42/69 (N=111)	10.60/12.88	0.89 (0.81–0.98)	0.0236	0.8122	*SHANK3*
ALL	ADHD	R-HSA-422475	Axon guidance	542	2	42/69 (N=111)	10.12/12.20	0.90 (0.81–1.00)	0.0472	0.8306	*SHANK3*
ALL	ASD	R-HSA-1655829	Regulation of cholesterol biosynthesis by SREBP (SREBF)	55	3	68/44 (N=112)	1.26/1.75	0.72 (0.54–0.96)	0.0275	0.8122	*TBL1XR1*
ALL	ID	R-HSA-5617472	Activation of anterior HOX genes in hindbrain development during early embryogenesis	91	2	49/63 (N=112)	0.69/1.37	0.53 (0.36–0.79)	0.0018	0.3528	*KMT2C*
ALL	ID	R-HSA-5619507	Activation of HOX genes during differentiation	91	1	49/63 (N=112)	0.69/1.37	0.53 (0.36–0.79)	0.0018	0.3528	*KMT2C*
ALL	ID	R-HSA-9772755	Formation of WDR5-containing histone-modifying complexes	42	3	49/63 (N=112)	0.73/1.30	0.59 (0.41–0.85)	0.0048	0.5207	*KMT2C*
ALL	ID	R-HSA-3247509	Chromatin modifying enzymes	238	1	47/63 (N=110)	3.49/4.90	0.74 (0.60–0.92)	0.0075	0.5491	*KAT6A*; *KMT2C*; *TBL1XR1*; *TRRAP*
ALL	ID	R-HSA-4839726	Chromatin organization	346	0	46/63 (N=109)	5.13/6.78	0.79 (0.65–0.96)	0.0164	0.6910	*ADNP*; *KAT6A*; *KMT2C*; *TBL1XR1*; *TCF4*; *TRRAP*
ALL	ID	R-HSA-3214815	HDACs deacetylate histones	60	2	49/63 (N=112)	0.45/0.84	0.56 (0.33–0.94)	0.0276	0.8122	*TBL1XR1*
ALL	ID	R-HSA-9938024	Somatic hypermutation of immunoglobulin genes	169	4	49/63 (N=112)	2.71/3.54	0.80 (0.65–1.00)	0.0476	0.8306	*KMT2C*
ALL	SLD	R-HSA-2672351	Stimuli-sensing channels	106	2	6/106 (N=112)	2.17/3.54	0.64 (0.41–0.98)	0.0408	0.8280	*CLCN4*
HC80	ADHD	R-HSA-9675108	Nervous system development	568	1	36/63 (N=99)	10.19/12.95	0.87 (0.78–0.97)	0.0103	0.7367	*SHANK3*
HC80	ADHD	R-HSA-422475	Axon guidance	542	2	36/63 (N=99)	9.69/12.27	0.87 (0.78–0.98)	0.0182	0.7367	*SHANK3*
HC80	ASD	R-HSA-9711123	Cellular response to chemical stress	204	2	61/39 (N=100)	3.10/3.90	0.79 (0.65–0.96)	0.0174	0.7367	*TBL1XR1*
HC80	ASD	R-HSA-1655829	Regulation of cholesterol biosynthesis by SREBP (SREBF)	55	3	61/39 (N=100)	1.16/1.64	0.70 (0.51–0.96)	0.0266	0.8019	*TBL1XR1*
HC80	DCD	R-HSA-9855142	Cellular responses to mechanical stimuli	103	1	8/93 (N=101)	0.75/2.03	0.39 (0.16–0.95)	0.0386	0.8019	*PRKAR1B*
HC80	ID	R-HSA-3214847	HATs acetylate histones	108	2	45/55 (N=100)	1.18/1.93	0.64 (0.46–0.91)	0.0120	0.7367	*TRRAP*
HC80	ID	R-HSA-3247509	Chromatin modifying enzymes	238	1	44/55 (N=99)	3.45/4.82	0.75 (0.60–0.94)	0.0141	0.7367	*TBL1XR1*; *TRRAP*
HC80	ID	R-HSA-4839726	Chromatin organization	346	0	43/55 (N=98)	5.14/6.75	0.80 (0.65–0.98)	0.0293	0.8019	*ADNP*; *TBL1XR1*; *TCF4*; *TRRAP*
HC80	SLD	R-HSA-2672351	Stimuli-sensing channels	106	2	6/94 (N=100)	2.17/3.53	0.64 (0.41–0.98)	0.0421	0.8019	*CLCN4*

Global total VUS burden among the patients with a VUS file (N=113): DSM-5 ID+ n=50, median 509 [IQR 489.25–543]; ID− n=63, median 511 [486–536]. Two-sided Mann–Whitney U=1609, p=0.8464. This comparison does not support global burden as the explanation for the inverse chromatin-pathway signals; it does not exclude pathway-specific confounding or sampling variability.

Table S13. Profile–diagnosis pairing and overlap among patients with a VUS file (before pathway-specific exclusions)

Class	Profile	Paired diagnosis	Rationale	ALL profile n	ALL dx n	ALL overlap	Jaccard	OR	p	q	HC80 overlap	HC80 Jaccard
1	Cognitive–Language	Sp-Lan-D	Highest prevalence within the profile and consistency with the cognitive–language phenotype.	29	80	23	0.267	1.816	0.1762	0.8034	19	0.25
2	Executive–Psychiatric	ADHD	High prevalence, enrichment, and clinical consistency with the executive–psychiatric phenotype.	26	43	19	0.38	7.125	4.188e-05	0.00101	15	0.349
3	Social–Sensory/Repetitive	ASD	High prevalence and enrichment, with relevance to social, sensory, and repetitive features.	38	69	30	0.39	3.462	0.00438	0.035	29	0.42
4	Motor–Neurological	DCD	Chosen for motor specificity and enrichment despite lower prevalence.	20	10	6	0.25	9.536	0.00199	0.0239	5	0.227

Table S14. HC80 exclusion characterization

Variable	Type	Excluded (n=14)	Retained (n=114)	Effect	p	q
Male sex	binary	12/14 (85.7%)	70/114 (61.4%)	OR 3.77	0.0845	1.0000
WES positive	binary	4/14 (28.6%)	22/114 (19.3%)	OR 1.67	0.4812	1.0000
WES still to discuss	binary	2/14 (14.3%)	7/114 (6.1%)	OR 2.55	0.2558	1.0000
Trio analysis	binary	12/14 (85.7%)	112/114 (98.2%)	OR 0.11	0.0589	1.0000
VUS file available	binary	12/14 (85.7%)	101/114 (88.6%)	OR 0.77	0.6688	1.0000
Diagnosis: ASD	binary	8/14 (57.1%)	72/114 (63.2%)	OR 0.78	0.7717	1.0000
Diagnosis: ADHD	binary	6/14 (42.9%)	40/114 (35.1%)	OR 1.39	0.5683	1.0000
Diagnosis: SLD	binary	0/14 (0.0%)	7/114 (6.1%)	OR 0.00	1.0000	1.0000
Diagnosis: Sp-Lan-D	binary	11/14 (78.6%)	79/114 (69.3%)	OR 1.62	0.5533	1.0000
Diagnosis: DCD	binary	2/14 (14.3%)	10/114 (8.8%)	OR 1.73	0.6203	1.0000
Diagnosis: ID	binary	6/14 (42.9%)	53/114 (46.5%)	OR 0.86	1.0000	1.0000
Symptom: Expressive language delay	binary	11/14 (78.6%)	90/114 (78.9%)	OR 0.98	1.0000	1.0000
Symptom: Delayed walking	binary	4/14 (28.6%)	37/114 (32.5%)	OR 0.83	1.0000	1.0000
Symptom: Developmental regression	binary	0/14 (0.0%)	21/114 (18.4%)	OR 0.00	0.1240	1.0000
Symptom: Intellectual disability	binary	9/14 (64.3%)	66/114 (57.9%)	OR 1.31	0.7773	1.0000
Symptom: Short attention span	binary	12/14 (85.7%)	90/114 (78.9%)	OR 1.60	0.7336	1.0000
Symptom: Hyperactivity	binary	8/14 (57.1%)	69/114 (60.5%)	OR 0.87	1.0000	1.0000
Symptom: Impulsivity	binary	7/14 (50.0%)	61/114 (53.5%)	OR 0.87	1.0000	1.0000
Symptom: Sensory processing abnormality	binary	8/14 (57.1%)	69/114 (60.5%)	OR 0.87	1.0000	1.0000
Symptom: Abnormal social interaction	binary	6/14 (42.9%)	68/114 (59.6%)	OR 0.51	0.2613	1.0000
Symptom: Motor stereotypy	binary	6/14 (42.9%)	55/114 (48.2%)	OR 0.80	0.7818	1.0000
Symptom: Restricted/repetitive behaviors	binary	6/14 (42.9%)	32/114 (28.1%)	OR 1.92	0.3515	1.0000
Symptom: Clumsiness	binary	5/14 (35.7%)	53/114 (46.5%)	OR 0.64	0.5730	1.0000
Symptom: Verbal dyspraxia	binary	3/14 (21.4%)	13/114 (11.4%)	OR 2.12	0.3826	1.0000
Symptom: Aggressive behavior	binary	8/14 (57.1%)	56/114 (49.1%)	OR 1.38	0.7781	1.0000

Variable	Type	Excluded (n=14)	Retained (n=114)	Effect	p	q
Symptom: Irritability	binary	5/14 (35.7%)	34/114 (29.8%)	OR 1.31	0.7595	1.0000
Symptom: Anxiety	binary	7/14 (50.0%)	41/114 (36.0%)	OR 1.78	0.3829	1.0000
Symptom: Psychotic episodes	binary	0/14 (0.0%)	1/114 (0.9%)	OR 0.00	1.0000	1.0000
Symptom: Obsessive-compulsive trait	binary	2/14 (14.3%)	6/114 (5.3%)	OR 3.00	0.2121	1.0000
Symptom: Abnormal emotional state	binary	0/14 (0.0%)	8/114 (7.0%)	OR 0.00	0.5971	1.0000
Symptom: Involuntary movements	binary	2/14 (14.3%)	10/114 (8.8%)	OR 1.73	0.6203	1.0000
Symptom: Ataxia	binary	0/14 (0.0%)	10/114 (8.8%)	OR 0.00	0.6000	1.0000
Symptom: Hearing impairment	binary	0/14 (0.0%)	5/114 (4.4%)	OR 0.00	1.0000	1.0000
Symptom: Pyramidal signs	binary	0/14 (0.0%)	10/114 (8.8%)	OR 0.00	0.6000	1.0000
Symptom: Extrapyramidal motor abnormality	binary	0/14 (0.0%)	2/114 (1.8%)	OR 0.00	1.0000	1.0000
Symptom: Hypotonia	binary	0/14 (0.0%)	9/114 (7.9%)	OR 0.00	0.5958	1.0000
Symptom: Seizures	binary	0/14 (0.0%)	16/114 (14.0%)	OR 0.00	0.2136	1.0000
Age at last clinical evaluation (years)	continuous	8.00 [6.25–13.25]	9.00 [7.00–12.00]	U 778.0	0.8804	1.0000
Retained symptom count (of 23)	continuous	8.50 [6.00–9.00]	8.00 [6.25–10.00]	U 737.5	0.6440	1.0000
Total filtered VUS (patients with file)	continuous	530.50 [505.25–552.25]	509.00 [487.00–536.00]	U 799.0	0.0728	1.0000

Table S15. Posterior-weighted sensitivity for the pathway-specific primary positive profile signal. Descriptive soft weights; formal BCH results are reported separately in Table S21.

Class	Profile	Pathway	Hard IRR	Hard p	Weighted rate ratio	Bootstrap 95% CI	Sign p	Interpretation
3	Social–Sensory/Repetitive	Transcriptional Regulation by *MECP2*	1.462	0.0223	1.420	1.042–1.909	0.0236	Descriptive soft-membership sensitivity; not a BCH/ML three-step estimator.

Table S16. Key sex- and age-adjusted checks for pathway-specific primary findings

Framework / group	Pathway	Eligible n	Primary IRR	Primary p	Sex/age IRR (95% CI)	Adjusted p	Interpretation
Profile C3	Transcriptional Regulation by *MECP2*	111	1.46	0.0223	1.40 (1.00–1.96)	0.0500	Positive direction retained; no longer nominal (exact p=0.050013).
Diagnosis ADHD	Metabolism	110	1.12	0.0009	1.06 (0.99–1.13)	0.0848	No longer nominal after sex/age adjustment.

Note: these estimates are computed on the pathway-specific primary cohorts of statistical-report worksheets 24 and 30 and are listed, for every primary comparison of both cohorts, in worksheet 61; worksheets 41 and 42 carry the same covariate models on the unfiltered cohort. Age is age at last clinical evaluation. No adjusted comparison is FDR-significant.

Table S16 (continued). Covariate-model family summary for the covariate sensitivity analysis on the unfiltered cohort

Framework	Cohort	Model	Adjustments	Positive p<0.05	Positive q<0.05	Inverse p<0.05	Median Poisson dispersion
profile	ALL	Poisson	Sex, age	1	0	2	1.1259
profile	ALL	Poisson	Sex, age, trio	2	0	3	1.1312
profile	ALL	Negative binomial	Sex, age	1	0	—	—
profile	ALL	Negative binomial	Sex, age, trio	2	0	—	—
profile	ALL	Logistic	Sex, age	1	0	—	—

Framework	Cohort	Model	Adjustments	Positive p<0.05	Positive q<0.05	Inverse p<0.05	Median Poisson dispersion
profile	ALL	Logistic	Sex, age, trio	0	0	—	—
profile	HC80	Poisson	Sex, age	2	0	1	1.1128
profile	HC80	Poisson	Sex, age, trio	2	0	4	1.0985
profile	HC80	Negative binomial	Sex, age	2	0	—	—
profile	HC80	Negative binomial	Sex, age, trio	2	0	—	—
profile	HC80	Logistic	Sex, age	2	0	—	—
profile	HC80	Logistic	Sex, age, trio	0	0	—	—
diagnosis	ALL	Poisson	Sex, age	34	0	10	1.1107
diagnosis	ALL	Poisson	Sex, age, trio	32	1	9	1.1112
diagnosis	ALL	Negative binomial	Sex, age	33	0	—	—
diagnosis	ALL	Negative binomial	Sex, age, trio	33	1	—	—
diagnosis	ALL	Logistic	Sex, age	9	0	—	—
diagnosis	ALL	Logistic	Sex, age, trio	6	0	—	—
diagnosis	HC80	Poisson	Sex, age	28	0	7	1.1036
diagnosis	HC80	Poisson	Sex, age, trio	26	0	9	1.0979
diagnosis	HC80	Negative binomial	Sex, age	28	0	—	—
diagnosis	HC80	Negative binomial	Sex, age, trio	26	0	—	—
diagnosis	HC80	Logistic	Sex, age	6	0	—	—
diagnosis	HC80	Logistic	Sex, age, trio	1	0	—	—

Full models additionally adjusted for trio status. Trio status is near-constant in the populations these models use — the unfiltered cohorts — where 109 of 113 patients were sequenced as trios (99 of 101 in HC80), leaving four and two non-trio patients respectively; the corresponding figure for the global informative-WES exclusion cohort, which these worksheets do not use, is 90 of 93. Where a full model is estimable and regular its group estimate keeps its usual interpretation; only the boundary cases, in which the trio indicator is nearly collinear with the group indicator, reduce to the contrast within the trio-sequenced patients. Across all comparisons the full and sex/age-adjusted group estimates differ by a median of 0.01 on the log scale and no interval widens by more than 25%. Inverse counts and dispersion were summarized only for Poisson models; dashes denote fields not included in this summary. Dispersion is displayed to four decimals; full precision is retained in the statistical workbook. Models with no estimate carry no p or q value and do not enter the Benjamini–Hochberg family.

Table S17. Leave-one-anchor-out summary

Framework	Cohort	Group	Removed anchor	Anchors before	Anchors after	Paths before	Paths after	Paths lost	Conv before	Conv after	Conv lost
profile	ALL	Cognitive–Language	*ANKRD11*	5	4	61	61	0	0	0	0
profile	ALL	Cognitive–Language	*KAT6A*	5	4	61	52	9	0	0	0
profile	ALL	Cognitive–Language	*PRKAR1B*	5	4	61	13	48	0	0	0
profile	ALL	Cognitive–Language	*SETBP1*	5	4	61	61	0	0	0	0

Framework	Cohort	Group	Removed anchor	Anchors before	Anchors after	Paths before	Paths after	Paths lost	Conv before	Conv after	Conv lost
profile	ALL	Cognitive–Language	*TRIP12*	5	4	61	57	4	0	0	0
profile	ALL	Executive–Psychiatric	*ADNP*	3	2	41	37	4	1	0	1
profile	ALL	Executive–Psychiatric	*ATRX*	3	2	41	30	11	1	1	0
profile	ALL	Executive–Psychiatric	*KMT2C*	3	2	41	16	25	1	0	1
profile	ALL	Social–Sensory/Repetitive	*CLCN4*	12	11	97	94	3	23	23	0
profile	ALL	Social–Sensory/Repetitive	*L1CAM*	12	11	97	92	5	23	21	2
profile	ALL	Social–Sensory/Repetitive	*LZTR1*	12	11	97	97	0	23	23	0
profile	ALL	Social–Sensory/Repetitive	*MBD5*	12	11	97	96	1	23	20	3
profile	ALL	Social–Sensory/Repetitive	*MED13L*	12	11	97	95	2	23	16	7
profile	ALL	Social–Sensory/Repetitive	*MYT1L*	12	11	97	97	0	23	23	0
profile	ALL	Social–Sensory/Repetitive	*NLGN1*	12	11	97	97	0	23	20	3
profile	ALL	Social–Sensory/Repetitive	*SCN2A*	12	11	97	91	6	23	21	2
profile	ALL	Social–Sensory/Repetitive	*SHANK3*	12	11	97	96	1	23	20	3
profile	ALL	Social–Sensory/Repetitive	*SMPD4*	12	11	97	94	3	23	23	0
profile	ALL	Social–Sensory/Repetitive	*TBL1XR1*	12	11	97	49	48	23	13	10
profile	ALL	Social–Sensory/Repetitive	*TRRAP*	12	11	97	92	5	23	17	6
profile	ALL	Motor–Neurological	*AUTS2*	7	6	25	23	2	4	1	3
profile	ALL	Motor–Neurological	*PHIP*	7	6	25	20	5	4	3	1
profile	ALL	Motor–Neurological	*PURA*	7	6	25	25	0	4	4	0
profile	ALL	Motor–Neurological	*RBFOX1*	7	6	25	23	2	4	1	3
profile	ALL	Motor–Neurological	*SRCAP*	7	6	25	25	0	4	4	0
profile	ALL	Motor–Neurological	*TCF4*	7	6	25	13	12	4	3	1
profile	ALL	Motor–Neurological	*TMEM240*	7	6	25	25	0	4	4	0
profile	HC80	Cognitive–Language	*ANKRD11*	3	2	52	52	0	0	0	0
profile	HC80	Cognitive–Language	*PRKAR1B*	3	2	52	4	48	0	0	0
profile	HC80	Cognitive–Language	*TRIP12*	3	2	52	48	4	0	0	0
profile	HC80	Executive–Psychiatric	*ADNP*	2	1	16	11	5	0	0	0
profile	HC80	Executive–Psychiatric	*ATRX*	2	1	16	5	11	0	0	0
profile	HC80	Social–Sensory/Repetitive	*CLCN4*	12	11	97	94	3	23	23	0
profile	HC80	Social–Sensory/Repetitive	*L1CAM*	12	11	97	92	5	23	21	2
profile	HC80	Social–Sensory/Repetitive	*LZTR1*	12	11	97	97	0	23	23	0
profile	HC80	Social–Sensory/Repetitive	*MBD5*	12	11	97	96	1	23	20	3
profile	HC80	Social–Sensory/Repetitive	*MED13L*	12	11	97	95	2	23	16	7
profile	HC80	Social–Sensory/Repetitive	*MYT1L*	12	11	97	97	0	23	23	0
profile	HC80	Social–Sensory/Repetitive	*NLGN1*	12	11	97	97	0	23	20	3
profile	HC80	Social–Sensory/Repetitive	*SCN2A*	12	11	97	91	6	23	21	2
profile	HC80	Social–Sensory/Repetitive	*SHANK3*	12	11	97	96	1	23	20	3
profile	HC80	Social–Sensory/Repetitive	*SMPD4*	12	11	97	94	3	23	23	0
profile	HC80	Social–Sensory/Repetitive	*TBL1XR1*	12	11	97	49	48	23	13	10
profile	HC80	Social–Sensory/Repetitive	*TRRAP*	12	11	97	92	5	23	17	6

Framework	Cohort	Group	Removed anchor	Anchors before	Anchors after	Paths before	Paths after	Paths lost	Conv before	Conv after	Conv lost
profile	HC80	Motor–Neurological	*AUTS2*	6	5	25	23	2	4	1	3
profile	HC80	Motor–Neurological	*PHIP*	6	5	25	20	5	4	3	1
profile	HC80	Motor–Neurological	*PURA*	6	5	25	25	0	4	4	0
profile	HC80	Motor–Neurological	*RBFOX1*	6	5	25	23	2	4	1	3
profile	HC80	Motor–Neurological	*TCF4*	6	5	25	13	12	4	3	1
profile	HC80	Motor–Neurological	*TMEM240*	6	5	25	25	0	4	4	0
diagnosis	ALL	ADHD	*ADNP*	10	9	134	130	4	23	23	0
diagnosis	ALL	ADHD	*ANKRD11*	10	9	134	134	0	23	23	0
diagnosis	ALL	ADHD	*KMT2C*	10	9	134	119	15	23	14	9
diagnosis	ALL	ADHD	*MBD5*	10	9	134	130	4	23	23	0
diagnosis	ALL	ADHD	*MED13L*	10	9	134	132	2	23	17	6
diagnosis	ALL	ADHD	*MYT1L*	10	9	134	134	0	23	23	0
diagnosis	ALL	ADHD	*PRKAR1B*	10	9	134	92	42	23	20	3
diagnosis	ALL	ADHD	*SHANK3*	10	9	134	129	5	23	22	1
diagnosis	ALL	ADHD	*SRCAP*	10	9	134	134	0	23	23	0
diagnosis	ALL	ADHD	*TBL1XR1*	10	9	134	95	39	23	6	17
diagnosis	ALL	ASD	*ATRX*	10	9	133	123	10	17	17	0
diagnosis	ALL	ASD	*AUTS2*	10	9	133	131	2	17	14	3
diagnosis	ALL	ASD	*L1CAM*	10	9	133	129	4	17	14	3
diagnosis	ALL	ASD	*LZTR1*	10	9	133	133	0	17	17	0
diagnosis	ALL	ASD	*MYT1L*	10	9	133	133	0	17	17	0
diagnosis	ALL	ASD	*NLGN1*	10	9	133	133	0	17	15	2
diagnosis	ALL	ASD	*PRKAR1B*	10	9	133	92	41	17	12	5
diagnosis	ALL	ASD	*SCN2A*	10	9	133	127	6	17	15	2
diagnosis	ALL	ASD	*SHANK3*	10	9	133	132	1	17	15	2
diagnosis	ALL	ASD	*TBL1XR1*	10	9	133	81	52	17	10	7
diagnosis	ALL	DCD	*PRKAR1B*	4	3	53	5	48	0	0	0
diagnosis	ALL	DCD	*RBFOX1*	4	3	53	48	5	0	0	0
diagnosis	ALL	DCD	*SETBP1*	4	3	53	53	0	0	0	0
diagnosis	ALL	DCD	*SRCAP*	4	3	53	53	0	0	0	0
diagnosis	ALL	ID	*ADNP*	17	16	168	166	2	28	26	2
diagnosis	ALL	ID	*ANKRD11*	17	16	168	168	0	28	28	0
diagnosis	ALL	ID	*ATRX*	17	16	168	158	10	28	28	0
diagnosis	ALL	ID	*AUTS2*	17	16	168	167	1	28	27	1
diagnosis	ALL	ID	*CLCN4*	17	16	168	166	2	28	27	1
diagnosis	ALL	ID	*KAT6A*	17	16	168	165	3	28	27	1
diagnosis	ALL	ID	*KMT2C*	17	16	168	156	12	28	20	8
diagnosis	ALL	ID	*PRKAR1B*	17	16	168	126	42	28	25	3
diagnosis	ALL	ID	*PURA*	17	16	168	168	0	28	28	0
diagnosis	ALL	ID	*RBFOX1*	17	16	168	167	1	28	27	1

Framework	Cohort	Group	Removed anchor	Anchors before	Anchors after	Paths before	Paths after	Paths lost	Conv before	Conv after	Conv lost
diagnosis	ALL	ID	*SCN2A*	17	16	168	158	10	28	28	0
diagnosis	ALL	ID	*SETBP1*	17	16	168	168	0	28	28	0
diagnosis	ALL	ID	*SMPD4*	17	16	168	165	3	28	27	1
diagnosis	ALL	ID	*TBL1XR1*	17	16	168	125	43	28	19	9
diagnosis	ALL	ID	*TCF4*	17	16	168	163	5	28	23	5
diagnosis	ALL	ID	*TRIP12*	17	16	168	166	2	28	26	2
diagnosis	ALL	ID	*TRRAP*	17	16	168	164	4	28	24	4
diagnosis	ALL	SLD	*CLCN4*	1	0	3	0	3	0	0	0
diagnosis	ALL	Sp-Lan-D	*ADNP*	20	19	112	110	2	19	17	2
diagnosis	ALL	Sp-Lan-D	*ANKRD11*	20	19	112	112	0	19	19	0
diagnosis	ALL	Sp-Lan-D	*ATRX*	20	19	112	102	10	19	18	1
diagnosis	ALL	Sp-Lan-D	*AUTS2*	20	19	112	110	2	19	16	3
diagnosis	ALL	Sp-Lan-D	*KAT6A*	20	19	112	109	3	19	14	5
diagnosis	ALL	Sp-Lan-D	*LZTR1*	20	19	112	112	0	19	19	0
diagnosis	ALL	Sp-Lan-D	*MBD5*	20	19	112	111	1	19	16	3
diagnosis	ALL	Sp-Lan-D	*MYT1L*	20	19	112	112	0	19	19	0
diagnosis	ALL	Sp-Lan-D	*NLGN1*	20	19	112	110	2	19	18	1
diagnosis	ALL	Sp-Lan-D	*PHIP*	20	19	112	107	5	19	19	0
diagnosis	ALL	Sp-Lan-D	*PRKAR1B*	20	19	112	68	44	19	16	3
diagnosis	ALL	Sp-Lan-D	*PURA*	20	19	112	112	0	19	19	0
diagnosis	ALL	Sp-Lan-D	*SCN2A*	20	19	112	102	10	19	18	1
diagnosis	ALL	Sp-Lan-D	*SETBP1*	20	19	112	112	0	19	19	0
diagnosis	ALL	Sp-Lan-D	*SMPD4*	20	19	112	108	4	19	18	1
diagnosis	ALL	Sp-Lan-D	*SRCAP*	20	19	112	112	0	19	19	0
diagnosis	ALL	Sp-Lan-D	*TCF4*	20	19	112	107	5	19	13	6
diagnosis	ALL	Sp-Lan-D	*TMEM240*	20	19	112	112	0	19	19	0
diagnosis	ALL	Sp-Lan-D	*TRIP12*	20	19	112	108	4	19	19	0
diagnosis	ALL	Sp-Lan-D	*TRRAP*	20	19	112	111	1	19	11	8
diagnosis	HC80	ADHD	*ADNP*	8	7	119	115	4	14	13	1
diagnosis	HC80	ADHD	*ANKRD11*	8	7	119	119	0	14	14	0
diagnosis	HC80	ADHD	*MBD5*	8	7	119	115	4	14	14	0
diagnosis	HC80	ADHD	*MED13L*	8	7	119	117	2	14	8	6
diagnosis	HC80	ADHD	*MYT1L*	8	7	119	119	0	14	14	0
diagnosis	HC80	ADHD	*PRKAR1B*	8	7	119	77	42	14	11	3
diagnosis	HC80	ADHD	*SHANK3*	8	7	119	114	5	14	13	1
diagnosis	HC80	ADHD	*TBL1XR1*	8	7	119	71	48	14	5	9
diagnosis	HC80	ASD	*ATRX*	10	9	133	123	10	17	17	0
diagnosis	HC80	ASD	*AUTS2*	10	9	133	131	2	17	14	3
diagnosis	HC80	ASD	*L1CAM*	10	9	133	129	4	17	14	3
diagnosis	HC80	ASD	*LZTR1*	10	9	133	133	0	17	17	0

Framework	Cohort	Group	Removed anchor	Anchors before	Anchors after	Paths before	Paths after	Paths lost	Conv before	Conv after	Conv lost
diagnosis	HC80	ASD	*MYT1L*	10	9	133	133	0	17	17	0
diagnosis	HC80	ASD	*NLGN1*	10	9	133	133	0	17	15	2
diagnosis	HC80	ASD	*PRKAR1B*	10	9	133	92	41	17	12	5
diagnosis	HC80	ASD	*SCN2A*	10	9	133	127	6	17	15	2
diagnosis	HC80	ASD	*SHANK3*	10	9	133	132	1	17	15	2
diagnosis	HC80	ASD	*TBL1XR1*	10	9	133	81	52	17	10	7
diagnosis	HC80	DCD	*PRKAR1B*	2	1	53	5	48	0	0	0
diagnosis	HC80	DCD	*RBFOX1*	2	1	53	48	5	0	0	0
diagnosis	HC80	ID	*ADNP*	14	13	153	151	2	19	17	2
diagnosis	HC80	ID	*ANKRD11*	14	13	153	153	0	19	19	0
diagnosis	HC80	ID	*ATRX*	14	13	153	143	10	19	19	0
diagnosis	HC80	ID	*AUTS2*	14	13	153	151	2	19	19	0
diagnosis	HC80	ID	*CLCN4*	14	13	153	151	2	19	18	1
diagnosis	HC80	ID	*PRKAR1B*	14	13	153	111	42	19	16	3
diagnosis	HC80	ID	*PURA*	14	13	153	153	0	19	19	0
diagnosis	HC80	ID	*RBFOX1*	14	13	153	152	1	19	18	1
diagnosis	HC80	ID	*SCN2A*	14	13	153	143	10	19	19	0
diagnosis	HC80	ID	*SMPD4*	14	13	153	150	3	19	18	1
diagnosis	HC80	ID	*TBL1XR1*	14	13	153	105	48	19	14	5
diagnosis	HC80	ID	*TCF4*	14	13	153	148	5	19	14	5
diagnosis	HC80	ID	*TRIP12*	14	13	153	149	4	19	19	0
diagnosis	HC80	ID	*TRRAP*	14	13	153	148	5	19	15	4
diagnosis	HC80	SLD	*CLCN4*	1	0	3	0	3	0	0	0
diagnosis	HC80	Sp-Lan-D	*ADNP*	17	16	109	107	2	14	12	2
diagnosis	HC80	Sp-Lan-D	*ANKRD11*	17	16	109	109	0	14	14	0
diagnosis	HC80	Sp-Lan-D	*ATRX*	17	16	109	99	10	14	13	1
diagnosis	HC80	Sp-Lan-D	*AUTS2*	17	16	109	104	5	14	14	0
diagnosis	HC80	Sp-Lan-D	*LZTR1*	17	16	109	109	0	14	14	0
diagnosis	HC80	Sp-Lan-D	*MBD5*	17	16	109	108	1	14	11	3
diagnosis	HC80	Sp-Lan-D	*MYT1L*	17	16	109	109	0	14	14	0
diagnosis	HC80	Sp-Lan-D	*NLGN1*	17	16	109	107	2	14	13	1
diagnosis	HC80	Sp-Lan-D	*PHIP*	17	16	109	104	5	14	14	0
diagnosis	HC80	Sp-Lan-D	*PRKAR1B*	17	16	109	65	44	14	11	3
diagnosis	HC80	Sp-Lan-D	*PURA*	17	16	109	109	0	14	14	0
diagnosis	HC80	Sp-Lan-D	*SCN2A*	17	16	109	99	10	14	13	1
diagnosis	HC80	Sp-Lan-D	*SMPD4*	17	16	109	105	4	14	13	1
diagnosis	HC80	Sp-Lan-D	*TCF4*	17	16	109	104	5	14	8	6
diagnosis	HC80	Sp-Lan-D	*TMEM240*	17	16	109	109	0	14	14	0
diagnosis	HC80	Sp-Lan-D	*TRIP12*	17	16	109	105	4	14	14	0
diagnosis	HC80	Sp-Lan-D	*TRRAP*	17	16	109	106	3	14	8	6

Table S18. Gene-level robustness summary for the pathway-specific primary C3 *MECP2* signal

Cohort	Profile	Pathway	Member genes	Observed genes tested	Primary IRR	Primary p	LOO positive direction	LOO nominal	Anchor dependency
ALL	Social–Sensory/Repetitive	Transcriptional Regulation by *MECP2*	63	42	1.462	0.0223	42/42	39/42	Pathway eligibility depends on *TBL1XR1* as the sole C3 anchor.

Note: the leave-one-observed-gene-out is computed on the pathway-specific primary cohort (n=111), the cohort of the primary IRR in this table, and is supplied in statistical-report worksheet "59 Gene LOO Primary". Forty-two of the 63 pathway members carry at least one VUS in that cohort; the positive direction is preserved after every removal and nominal significance after 39 of 42 (not retained after removing HIPK2, NCOR2 or TNRC6C). Worksheets 50 and 51 hold the same analysis on the unfiltered cohort (n=113), where BDNF also carries events and 43 genes are therefore tested, 40 of 43 retaining nominal significance.

Table S19. Approximate 80%-power minimum detectable effects for the global informative-WES exclusion cohort

Framework	Cohort	Group	Pathway	Case n	Control n	Observed IRR	MDE IRR	Observed carrier OR	MDE carrier OR	Assumption
profile	ALL	Social–Sensory/Repetitive	Transcriptional Regulation by *MECP2*	29	64	1.488	1.739	3.055	4.349	See note.
profile	HC80	Social–Sensory/Repetitive	Transcriptional Regulation by *MECP2*	27	57	1.580	1.794	3.119	4.489	See note.
diagnosis	ALL	ADHD	Metabolism	36	57	1.144	1.090	—	—	See note.
diagnosis	ALL	ADHD	Metabolism of lipids	36	57	1.240	1.132	—	—	See note.
diagnosis	ALL	ADHD	Circadian clock	36	57	1.394	1.544	5.611	7.138	See note.
diagnosis	ALL	ADHD	Adipogenesis	36	57	1.409	1.458	0.642	—	See note.
diagnosis	ALL	ADHD	Disorders of Developmental Biology	36	57	2.109	2.507	1.957	3.341	See note.
diagnosis	ALL	ADHD	Regulation of *MECP2* expression and activity	36	57	1.677	1.993	1.735	3.303	See note.
diagnosis	ALL	ADHD	Transcriptional Regulation by *MECP2*	36	57	1.460	1.700	1.266	4.885	See note.
diagnosis	ALL	ADHD	Viral Infection Pathways	36	57	1.120	1.159	—	—	See note.
diagnosis	ALL	ADHD	Cellular response to chemical stress	36	57	1.240	1.377	—	—	See note.
diagnosis	ALL	ASD	Viral Infection Pathways	61	32	1.144	1.161	—	—	See note.
diagnosis	ALL	DCD	Factors involved in megakaryocyte development and platelet production	7	86	1.548	1.778	—	—	See note.
diagnosis	ALL	ID	Vasopressin regulates renal water homeostasis via Aquaporins	38	55	2.338	2.324	2.635	3.280	See note.
diagnosis	ALL	ID	PKA activation in glucagon signalling	38	55	2.204	2.561	2.913	3.424	See note.
diagnosis	ALL	ID	Glucagon signaling in metabolic regulation	38	55	2.122	2.443	2.616	3.318	See note.
diagnosis	ALL	ID	Leishmania infection	38	55	1.346	1.323	—	—	See note.
diagnosis	ALL	ID	Parasitic Infection Pathways	38	55	1.346	1.323	—	—	See note.
diagnosis	ALL	ID	Aquaporin-mediated transport	38	55	1.864	2.148	2.092	3.203	See note.
diagnosis	ALL	ID	*ADORA2B* mediated anti-inflammatory cytokines production	38	55	2.057	2.346	2.358	3.280	See note.
diagnosis	ALL	ID	PKA-mediated phosphorylation of *CREB*	38	55	2.067	2.529	2.610	3.365	See note.
diagnosis	ALL	ID	PKA activation	38	55	2.067	2.529	2.610	3.365	See note.

Framework	Cohort	Group	Pathway	Case n	Control n	Observed IRR	MDE IRR	Observed carrier OR	MDE carrier OR	Assumption
diagnosis	ALL	ID	Anti-inflammatory response favouring Leishmania parasite infection	38	55	1.467	1.441	2.367	6.869	See note.
diagnosis	ALL	ID	Leishmania parasite growth and survival	38	55	1.467	1.441	2.367	6.869	See note.
diagnosis	ALL	SLD	Transport of small molecules	6	87	1.151	1.320	—	—	See note.
diagnosis	HC80	ADHD	Metabolism	32	52	1.166	1.096	—	—	See note.
diagnosis	HC80	ADHD	Metabolism of lipids	32	52	1.282	1.142	—	—	See note.
diagnosis	HC80	ADHD	Circadian clock	32	52	1.442	1.589	5.562	7.145	See note.
diagnosis	HC80	ADHD	Regulation of insulin secretion	32	52	1.459	1.750	2.075	5.732	See note.
diagnosis	HC80	ADHD	Integration of energy metabolism	32	52	1.340	1.542	2.245	—	See note.
diagnosis	HC80	ADHD	Adipogenesis	32	52	1.378	1.498	0.618	—	See note.
diagnosis	HC80	ADHD	Viral Infection Pathways	32	52	1.121	1.169	—	—	See note.
diagnosis	HC80	DCD	Factors involved in megakaryocyte development and platelet production	7	77	1.565	1.785	—	—	See note.
diagnosis	HC80	ID	Vasopressin regulates renal water homeostasis via Aquaporins	36	48	2.229	2.483	2.665	3.482	See note.
diagnosis	HC80	ID	Leishmania infection	36	48	1.357	1.338	—	—	See note.
diagnosis	HC80	ID	Parasitic Infection Pathways	36	48	1.357	1.338	—	—	See note.
diagnosis	HC80	ID	Anti-inflammatory response favouring Leishmania parasite infection	36	48	1.497	1.464	2.348	7.637	See note.
diagnosis	HC80	ID	Leishmania parasite growth and survival	36	48	1.497	1.464	2.348	7.637	See note.
diagnosis	HC80	SLD	Transport of small molecules	6	78	1.171	1.324	—	—	See note.
diagnosis	HC80	Sp-Lan-D	Aquaporin-mediated transport	59	25	2.162	2.413	2.025	3.695	See note.
diagnosis	HC80	Sp-Lan-D	Signaling by WNT	59	25	1.272	1.358	—	—	See note.
diagnosis	HC80	Sp-Lan-D	PKA activation in glucagon signalling	59	25	2.422	2.893	2.727	3.827	See note.

Note: MDE calculations use a normal approximation, two-sided α=0.05, Poisson variance without overdispersion, and are design diagnostics only. The case and control counts above are those of the global informative-WES exclusion cohort (n=93; HC80 n=84); statistical-report worksheet 52 reports the same diagnostics on the unfiltered cohort (n=113; HC80 n=101), so its counts and estimates differ from those given here.

Table S20. Reproducibility and quality-control summary for the pathway-specific primary analysis

Check	Expected	Observed	Status	Detail
Screened referrals	1104	1104	PASS	January 2024–February 2026.
Patients who underwent both CMA and WES	521	521	PASS	
Patients with comprehensive clinical evaluation	343	343	PASS	Manual-review population.
Manual-review exclusions (mutually exclusive)	215	215	PASS	60 + 80 + 17 + 24 + 31 + 3 = 215.
Excluded: diagnosis outside study eligibility	60	60	PASS	Includes perinatal brain injury, severe structural brain malformations, or isolated ID without additional NDD features.
Excluded: age criterion not met	80	80	PASS	

Excluded: WES data unavailable	17	17	PASS	
Excluded: clinical data unavailable	24	24	PASS	
Excluded: WES report not yet received at data lock	31	31	PASS	
Excluded: pathogenic/likely pathogenic CMA finding	3	3	PASS	
Unique clinical patients	128	128	PASS	Final analytical cohort; 343 − 215 = 128.
Patients with a VUS file	113	113	PASS	Starting set of the pathway-specific primary analysis.
Patients without a VUS file	15	15	PASS	
Patients with a VUS file: WES-negative status=0	84	84	PASS	
Patients with a VUS file: clinically informative status=1	20	20	PASS	Dynamically excluded only when the informative gene belongs to the pathway being tested.
Patients with a VUS file: unresolved status=2	9	9	PASS	Retained in pathway-specific primary tests; did not supply anchors.
HC80 patients overall	114	114	PASS	
HC80 patients with a VUS file	101	101	PASS	HC80 patients with a VUS file.
Pathway-specific primary eligible N range	107–113	107–113	PASS	Varies by tested pathway according to informative-WES gene membership.
Pathway-specific HC80 eligible N range	96–101	96–101	PASS	Applied within the 101 HC80 patients with a VUS file.
Global informative-WES exclusion sensitivity	93	93	PASS	Fixed sensitivity; HC80 n=84. No longer the primary outcome cohort.
Strict WES0 sensitivity	84	84	PASS	Fixed sensitivity; HC80 n=77.
Strict VUS after dedup	59950	59950	PASS	Count after declared quality/frequency filters and within-patient deduplication.
Patient totals reconcile to strict VUS	59950	59950	PASS	
Within-patient dedup key duplicates	0	0	PASS	
Diagnosis anchor pathways	183	183	PASS	
Profile pathway-specific primary comparisons	224	224	PASS	Poisson primary family; 4 nominal p<0.05, 0 q<0.05.
Diagnosis pathway-specific primary comparisons	603	603	PASS	Poisson primary family; 32 nominal p<0.05 (21 positive, 11 inverse), 0 q<0.05.
Profile primary Poisson FDR-positive rows	0	0	PASS	Pathway-specific primary family.
Diagnosis primary Poisson FDR-positive rows	0	0	PASS	Pathway-specific primary family.
Patient ID export	113	113	PASS	Patient-level export of the patients with a VUS file.
Intellectual disability flag	DX_ID present	DX_ID	PASS	
Unfiltered-cohort audit patients	113 / 101 HC80	113 / 101 HC80	PASS	ALL_LINKED and HC80_LINKED are explicitly present in the statistical workbook.
Global informative-WES exclusion patients	93 / 84 HC80	93 / 84 HC80	PASS	Global-exclusion rows computed independently from

				the patient-level data.
No-outlier patients	106 / 94 HC80	106 / 94 HC80	PASS	Tukey upper fence=613 VUS; 7/113 excluded, all HC80.
HC80 no-outlier profile Poisson FDR-positive rows	1	1	PASS	Inverse C1 Signaling by Hedgehog: IRR 0.57; p≈0.00023; q≈0.042.
Profile primary logistic FDR-positive rows	0	0	PASS	Adjusted-logistic carrier family after exclusion of separation-affected models.
Diagnosis primary logistic FDR-positive rows	0	0	PASS	Adjusted-logistic carrier family after exclusion of separation-affected models.
Separation-affected logistic models withdrawn	all flagged	30 profile; 98 diagnosis	PASS	Complete/quasi separation; reported as unavailable and excluded from the logistic BH family. Poisson, Mann-Whitney, Fisher and BCH endpoints are unaffected.

Component	Version
Python	3.12.13
Platform	Linux-6.18.35-x86_64-with-glibc2.39
pandas	2.2.3
numpy	2.3.5
scipy	1.17.0
scikit-learn	1.8.0
statsmodels	not installed; HC3 covariance computed directly in the frozen analysis pipeline
openpyxl	3.1.5
matplotlib	3.10.8
BCH / LCA-refit bootstrap	Python 3.12.14; NumPy 2.3.5; SciPy 1.17.0. Custom BCH equations and Bernoulli EM; no Mplus execution.
Population frequency source	gnomAD v4.1 via enGenome eVAI v3.1

Table S21. Formal BCH sensitivity of primary profile–pathway burden comparisons

The comparison below concerns C3 and Transcriptional Regulation by *MECP2* (R-HSA-8986944), a 63-gene pathway, with the same 111 eligible patients as the primary model. The reference classification is the 23-indicator K=4 clinical LCA fitted to the 128-patient cohort. All 224 primary profile comparisons, including nonsignificant and non-estimable results, are supplied in worksheet “55 BCH Primary”.

Inference	Rate ratio (95% CI)	Two-sided p	BH q
Primary hard assignment, Poisson HC3	1.462 (1.056–2.025)	0.0223	0.921
S15 descriptive posterior weights	1.420 (1.042–1.909)	0.0236*	Not calculated
Formal BCH, conditional patient-cluster HC1	1.515 (1.062–2.161)	0.0220	0.952

Inference	Rate ratio (95% CI)	Two-sided p	BH q
Formal BCH, LCA-refit bootstrap SE	1.515 (0.811–2.830)	0.1929	1.000

*S15 reports a descriptive bootstrap sign p value and 5,000 replicates. The BCH bootstrap uses 1,000 patient resamples, refits the LCA and yields a bootstrap standard error for log-IRR; the displayed BCH p value and interval use the normal-Wald approximation. The BCH percentile 95% interval for C3 *MECP2* is 0.576–2.027 and also includes 1. All 1,000 C3 *MECP2* bootstrap estimates were admissible.

BCH construction. For latent class k and modal class j, the classification-error matrix H(k,j) was the posterior-weighted proportion assigned to j. A patient assigned to j received row j of inverse(H) as the four signed BCH weights [1]. For the focal profile, case and pooled-comparator weights were w and 1−w. Weighted pathway-event sums divided by weighted total-VUS sums estimated the two Poisson rates; their ratio is the BCH IRR. The third-step clinical measurement parameters were fixed within each analyzed sample. Negative correction weights were retained. Inference was unavailable if either corrected event or exposure sum was nonpositive. Conditional covariance used patient-level joint scores, with HC1 factor N/(N−2), to account for the two weighted contributions from each patient.

Bootstrap specification. Entire patients were resampled from the 128-person clinical cohort, preserving their VUS outcomes and pathway eligibility. The 23-indicator K=4 Bernoulli LCA was re-estimated in each replicate with 40 starts (the observed-sample parameter values, two Gaussian perturbations with SD 0.08, and 37 random soft initializations), a 5,000-iteration limit, relative log-likelihood tolerance 10^{-9}, and probability bounds [10^{-7}, $1-10^{-7}$]. The best converged solution was retained. Hungarian matching aligned classes using mean squared differences in clinical item probabilities; genetic outcomes did not determine alignment. H and the BCH rate ratios were recomputed. Seed=20260913. This propagates measurement-model estimation uncertainty [2], conditional on the selected K, indicators, anchor/pathway universe and exclusion rules.

Validity and multiplicity. The observed H was well conditioned (condition number 1.141); signed weights ranged from −0.0663 to 1.0999. Of 224 comparisons, 218 yielded admissible point estimates. All 1,000 clinical bootstrap fits converged. Bootstrap inference was reported only with at least 950 admissible estimates: 216 comparisons met this criterion. CREB1 phosphorylation through the activation of Adenylate Cyclase and DARPP-32 events in C1 had 905 and 947 valid estimates. Six observed-sample comparisons were not estimable because of nonpositive corrected event sums. Separate BH families retained all 224 hypotheses for conditional and bootstrap inference; unavailable tests entered the correction internally as p=1 and remained explicitly unavailable in the results. Five conditional BCH comparisons were nominal, and none survived FDR. No comparison with reportable LCA-refit bootstrap inference was nominal or FDR-significant. Worksheet “57 BCH Bootstrap” reports replicate diagnostics. For C3–*MECP2*, the BCH point estimate is positive, but the LCA-refit bootstrap interval includes 1 (IRR 1.51, 95% CI 0.81–2.83; p=0.193). This supports an exploratory interpretation of the pathway-level signal and does not establish an association with *MECP2* alone.

Table S22. Coverage and interpretation of the external CDS and mutation-reference table

Reference field	All catalog symbols	Genes with events in worksheet 49
Total symbols / genes	11,106	4,565
MANE Select v1.5 CDS length available	10,428 / 11,106 (93.9%)	4,517 / 4,565 (98.9%)
Samocha 2014 mutation probabilities available	10,209 / 11,106 (91.9%)	4,470 / 4,565 (97.9%)

Note: gene-level contribution was compared with these annotations for the pathway-specific primary C3 / Transcriptional Regulation by MECP2 comparison (37 cases, 74 controls, 42 observed genes; statistical-report worksheet 60). Total gene-level VUS counts correlate with MANE CDS length (Spearman ρ=0.50, p=0.0007; Pearson r=0.69, p<0.0001) and with the Samocha missense mutability (ρ=0.50, p=0.0007), as expected of a length- and opportunity-driven count. No correlation was detected between the C3 excess and either annotation: ρ=-0.06, p=0.72 against CDS length and ρ=-0.04, p=0.80 against mutability. With 42 genes this absence of correlation does not exclude confounding by mutation opportunity. The excess is a difference of mean per-patient counts — gene-level events divided by the number of patients in each group — and is not normalized by each patient total VUS as the Poisson offset is. These are gene-level correlations, not a burden model adjusted for individual sequencing opportunity, which the available data do not support.

The complete table is supplied in Gene_CDS_Mutability_Reference.xlsx and statistical-report worksheet “58 Gene Reference”. Because Reactome pathways of infectious disease carry non-human participants, 374 of the 11,106 catalog symbols are viral or bacterial gene products — among them the SARS-CoV-2 accessory genes named 3A, 6, 7A, 8 and 9B — or RNA species; none was resolved to a human HGNC identifier, none carries an observed VUS, and they enter only the declared member-gene counts of the broad infection and immune pathways. It includes gene/HGNC identifiers, versioned MANE Select RefSeq and Ensembl transcripts, GRCh38.p14 CDS lengths [3], the Samocha source transcript and CDS/covered lengths, and synonymous, missense, nonsense, splice and frameshift mutation probabilities [4]. The loss-of-function aggregate is nonsense + splice + frameshift, and the protein-altering aggregate adds missense. Values are reference probabilities per gene copy per generation, not expected numbers of VUS in these patients or gene-constraint scores. The Samocha source coverage adjustment does not substitute for patient-specific callable sequence. Patient variants were aligned to GRCh37/hg19, whereas the MANE Select annotations are GRCh38.p14 transcript records; the two are joined by gene identity (HGNC symbol and identifier) and not by genomic coordinate, so no coordinate remapping of patient variants was performed or required (statistical-report worksheet “56 BCH Methods”, transcript-compatibility row).

The original Samocha source table was retrieved from the authors’ denovolyzeR repository at commit badc99f8d69e81742b94c409f072448f0f2fcfae (data-raw/fixed_mut_prob_fs_adjdepdiv.txt); source log10 values were converted to linear probabilities. HGNC symbol/identity matching was conservative. Missing or ambiguous references remain unavailable; the *PTPN20* mutation match was not forced. Where the source contained missing splice probability, conversion to zero followed the original authors’ parsing script and is flagged separately. Source URLs, hashes and mapping exceptions are documented in the spreadsheets.

Worksheet “49 Gene Decomp” supplies gene event counts for 42 unfiltered-cohort sensitivity contrasts and nine CDS/mutability reference columns. The separate reference workbook also includes all catalog members for those contrasts: 19,477 contrast–gene rows, including 7,153 verified zero-event rows. These records describe the unfiltered cohort rather than pathway-specific primary eligibility. The contribution–CDS/mutability correlation for the principal profile signal is reported separately in worksheet 60, computed on the pathway-specific primary cohort; gene-adjusted patient burden models are not included, because per-patient callable sequence is not available.

Table S23. Transcriptional Regulation by *MECP2 in both frameworks:* profile C3 against diagnosis ADHD on identical patients

Cohort	Model	Grouping	Cases	IRR	95% CI	p	Reference / note
ALL (n=111)	Marginal	Profile C3	37	1.46	1.06–2.03	0.0223	all other profiles
	Marginal	Diagnosis ADHD	42	1.42	1.03–1.97	0.0340	all patients without ADHD
	Marginal	Diagnosis ASD (paired with C3)	68	0.81	0.58–1.13	0.208	all patients without ASD
	Mutually adjusted	Profile C3	37	1.42	1.04–1.96	0.0296	C3 and ADHD in one model
	Mutually adjusted	Diagnosis ADHD	42	1.38	1.01–1.90	0.0442	C3 and ADHD in one model
	Cross-classified	C3 only	21	1.35	0.88–2.09	0.172	reference: neither (n=48)
	Cross-classified	ADHD only	26	1.32	0.86–2.01	0.199	reference: neither (n=48)
	Cross-classified	C3 and ADHD	16	2.00	1.26–3.17	0.0034	reference: neither (n=48)
	Difference	C3 versus ADHD	—	1.03	0.66–1.59	0.900	ratio of the two IRRs in the joint model

Cohort	Model	Grouping	Cases	IRR	95% CI	p	Reference / note
	Interaction	C3 × ADHD	—	1.12	0.58–2.15	0.735	departure from a multiplicative combination
HC80 (n=99)	Marginal	Profile C3	35	1.53	1.07–2.18	0.0188	all other profiles
	Marginal	Diagnosis ADHD	36	1.42	0.99–2.05	0.0589	all patients without ADHD
	Marginal	Diagnosis ASD (paired with C3)	61	0.76	0.52–1.09	0.133	all patients without ASD
	Mutually adjusted	Profile C3	35	1.48	1.05–2.10	0.0267	C3 and ADHD in one model
	Mutually adjusted	Diagnosis ADHD	36	1.37	0.96–1.95	0.0829	C3 and ADHD in one model
	Cross-classified	C3 only	20	1.40	0.88–2.21	0.156	reference: neither (n=43)
	Cross-classified	ADHD only	21	1.28	0.78–2.10	0.330	reference: neither (n=43)
	Cross-classified	C3 and ADHD	15	2.05	1.24–3.39	0.0049	reference: neither (n=43)
	Difference	C3 versus ADHD	—	1.08	0.67–1.77	0.745	ratio of the two IRRs in the joint model
	Interaction	C3 × ADHD	—	1.15	0.56–2.36	0.703	departure from a multiplicative combination

Note: the pathway VUS count is modeled with log(total filtered VUS) as offset and HC3 standard errors, as in Tables S10 to S12. On the pathway-specific primary cohorts the marginal rows reproduce those tables exactly, and the unfiltered cohorts reproduce the circularity_audit_unfiltered rows of worksheets 24 and 30; the outlier-trimmed cohorts of worksheet 62 keep the pathway-specific circularity exclusion (n=104 and 92) and are therefore not the ALL_NO_OUTLIERS / HC80_NO_OUTLIERS scopes of those worksheets (n=106 and 94), so their estimates are not expected to match. The two labels identify largely different patients: 16 of the 63 labeled patients carry both in ALL (Jaccard 0.25, odds ratio 1.41, Fisher p=0.42) and 15 of 56 in HC80 (0.27, 1.54, p=0.38), although a non-significant overlap test does not establish independence. Each estimate remains nominal after adjustment for the other in ALL, whereas in HC80 only C3 does (ADHD p=0.083). The two frameworks were not shown to differ: the Wald test of the difference between their coefficients in the joint model gives a ratio of 1.03 (95% CI 0.66–1.59; p=0.90) in ALL and 1.08 (0.67–1.77; p=0.75) in HC80, so the fact that C3 reaches p<0.05 in HC80 while ADHD does not is a difference in significance and not a demonstrated difference in effect. The C3 × ADHD interaction is likewise not significant (p=0.74 in ALL, 0.70 in HC80), so the estimate for patients carrying both labels is compatible with a multiplicative combination of the two. Both estimates attenuate under sex/age adjustment (C3 p=0.053, ADHD p=0.063 in ALL) and under full adjustment, and in the outlier-trimmed cohorts. Dispersion ranges from 0.85 to 1.04 across all models and from 0.86 to 0.98 in the joint models. Reproducibility: all models come from a single analysis script (xframework.py, available from the corresponding author on request) with numpy default_rng seed 20260901; the bootstrap draws 2,000 nonparametric resamples of patients with replacement and reports percentile intervals, discarding resamples in which either label is constant; the permutation test permutes the tested label 20,000 times with the other term held fixed and reports a two-sided p on |z| as (k+1)/(m+1). The pathway was selected because it was already nominal in both frameworks, so this comparison is conditioned on that selection: it is descriptive, carries no q value, enters no Benjamini–Hochberg family and supports no confirmatory claim. Neither comparison is FDR-significant in its own family. Statistical-report worksheet 62 carries every model, cohort and diagnostic label.

Supplementary Data Files

The accompanying Statistical_Report.xlsx is an integral machine-readable supplement. Worksheets “24 Profile Results All” and “30 Dx Results All” list the pathway-specific primary ALL and HC80 results first, followed by global-exclusion, strict WES-negative, no-outlier and unfiltered-cohort analyses labeled as sensitivities or audit rows. Worksheets “25 Profile Families” and “31 Dx Families” provide endpoint-level multiplicity counts. Supplementary-Patient_Level_Report.xlsx documents the 113 patients with a VUS file and the pathway-specific exclusion of informative-WES patients. Worksheets “55 BCH Primary”, “56 BCH Methods” and “57 BCH Bootstrap” contain the formal sensitivity, specifications and replicate diagnostics; “58 Gene Reference” supplies external CDS/mutability annotations. Patient-report worksheet “14 BCH Weights” contains signed correction weights for all 128 clinical patients and identifies the patients with a VUS file. Gene_CDS_Mutability_Reference.xlsx supplies the complete external-reference table and its gene-level linkage to the unfiltered-cohort contrasts. BCH source code and input specifications are listed with their checksums in statistical-report worksheet “06 File Manifest” and are available from the corresponding author on request; individual-level inputs are subject to the manuscript’s data-sharing restrictions.

References

1. Asparouhov T, Muthén B. Auxiliary Variables in Mixture Modeling: Using the BCH Method in Mplus to Estimate a Distal Outcome Model and an Arbitrary Secondary Model. Mplus Web Notes No. 21, version 11. 2021 Feb 4. Available from: https://www.statmodel.com/examples/webnotes/webnote21.pdf.
2. Bakk Z, Kuha J. Relating latent class membership to external variables: an overview. Br J Math Stat Psychol. 2021;74(2):340–362. doi:10.1111/bmsp.12227.
3. Morales J, Pujar S, Loveland JE, et al. A joint NCBI and EMBL-EBI transcript set for clinical genomics and research. Nature. 2022;604(7905):310–315. doi:10.1038/s41586-022-04558-8. MANE Select v1.5 data: https://www.ncbi.nlm.nih.gov/refseq/MANE/ (accessed 13 September 2026).
4. Samocha KE, Robinson EB, Sanders SJ, et al. A framework for the interpretation of de novo mutation in human disease. Nat Genet. 2014;46(9):944-950. doi:10.1038/ng.3050.
